# Supplementary material for: Novel Green Fluorescent Polyamines to Analyze ATP13A2 and ATP13A3 Activity in the Mammalian Polyamine Transport System
Source: Biomolecules. 2023 Feb 9;13(2):337. doi: 10.3390/biom13020337 (PMC9953717; doi:10.3390/biom13020337)
Supplement: Supplementary file 1 [file biomolecules-13-00337-s001.zip › biomolecules-2132768-supplementary.pdf]

Supplementary information for:

Novel green fluorescent polyamines to analyze ATP13A2 and ATP13A3 activity in the mammalian polyamine transport system

Marine Houdou[1,2,†], Nathalie Jacobs[1,†], Jonathan Coene[3,†], Mujahid Azfar[1], Roeland Vanhoutte[3], Chris Van den Haute[2,4], Jan Eggermont[1], Veronique Daniëls[2,5], Steven H. L. Verhelst[3,‡,*] and Peter Vangheluwe[1,2, ‡,*].

1. Laboratory of Cellular Transport Systems, Department of Cellular and Molecular Medicine, KU Leuven, B 3000 Leuven, Belgium
2. Aligning Science Across Parkinson's (ASAP) Collaborative Research Network, KU Leuven, B 3000 Leuven, Belgium
3. Laboratory of Chemical Biology, Department of Cellular and Molecular Medicine, KU Leuven, B 3000 Leuven, Belgium
4. Leuven Viral Vector Core, KU Leuven, Leuven, Belgium.
5. Research Group for Neurobiology and Gene Therapy, Department of Neurosciences, KU Leuven, B 3000 Leuven, Belgium

†, ‡ These authors contributed equally to this work.

* Correspondence: steven.verhelst@kuleuven.be; +32 16 37 45 17; SV and peter.vangheluwe@kuleuven.be; +32 16 33 07 20; PV.

This file contains:

Supplementary synthetic methods and schemes
Supplementary ^{1}H-NMR and ^{13}C-NMR and HRMS data
Supplementary LC-chromatograms of final compounds including purity
Supplementary Figures with data

Synthetic methods

All starting materials and solvents were purchased from commercial vendors and used without further purification unless specified otherwise. Raney®-Nickel was purchased as a 50% slurry in water from Acros Organics (catalog number 395921000). Reactions were analysed by Thin Layer Chromatography (TLC) on pre-coated 0.20 mm thick ALUGRAM® TLC sheets with fluorescent indicator and by liquid chromatography mass spectrometry (LC MS) performed on a Prominence Ultra fast Liquid Chromatography system equipped with a 2x150 mm C18 analytical column (Waters X Bridge) coupled to a MS-2020 single quadrupole mass analyser (Shimadzu). A linear gradient of 5 to 90% acetonitrile in water (containing 0.1% formic acid) was used. Purity of compounds was assessed using 215 nm HPLC trace. The injection peak and peaks determined to be baseline variations that appear in all spectra were removed manually. HRMS spectra were acquired on a quadrupole orthogonal acceleration time of flight mass spectrometer (Synapt G2 HDMS, Waters, Milford, MA). Samples were infused at 3μL/min and spectra were obtained in positive ionization mode with a resolution of 15000 (FWHM) using leucine enkephalin as lock mass. Silica column chromatography was performed using 230-400 mesh silica (Kieselgel 60). NMR spectra were recorded in deuterated solvents as indicated and measured using a Bruker UltraShield 600 MHz NMR spectrometer. Chemical shifts are reported in ppm relative to the residual solvent peak and J-values are reported in Hertz.

The azide precursors were synthesized based on previously described procedures [1,2]. The symmetrical BODIPY probes were synthesized as HCl salts based on previously described procedures [1, 2].

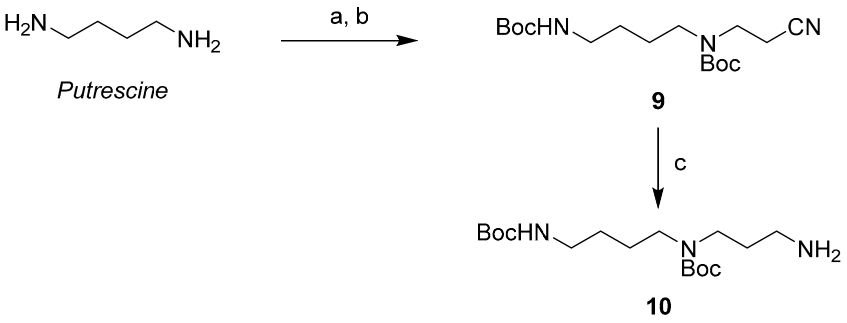

Figure S1. Synthesis of putrescine precursors, reagents and conditions: (a) Acrylonitrile, Et_3N, MeOH, 0 °C → rt. (b) Boc_2O, Et_3N, MeOH, 0 °C → rt. (c) Raney®-Nickel, NaOH, 2.3 bar of H_2, MeOH, rt.

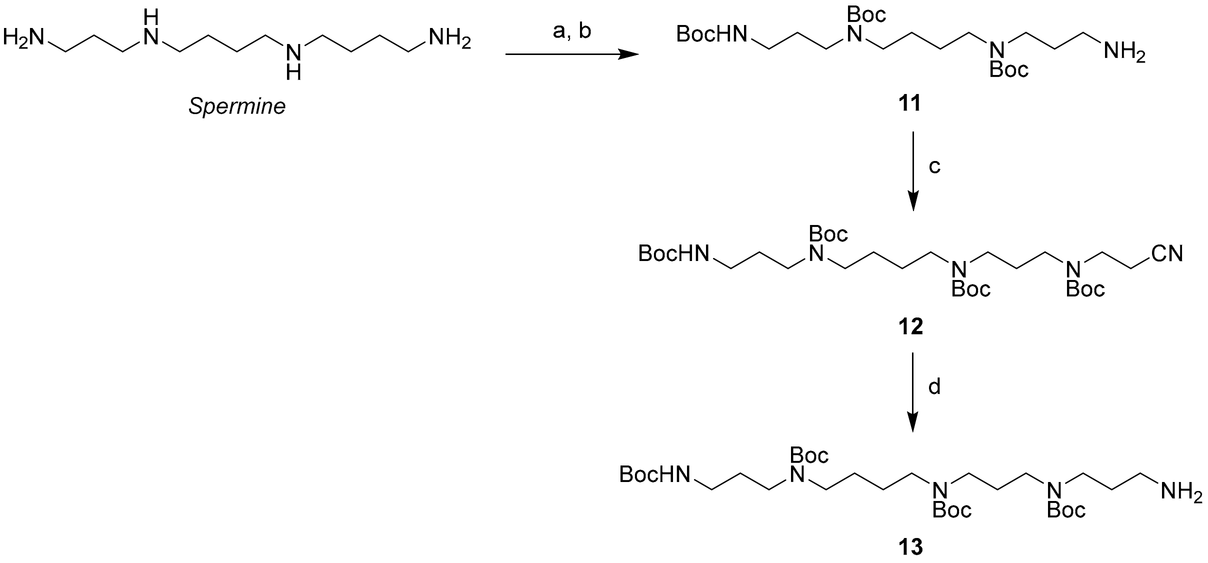

Figure S2. Synthesis of spermidine and spermine precursors, reagents and conditions: (a) EtTFA, MeOH, 0 °C → rt., then Boc_2O. (b) $NaOH_{(aq)}$, MeOH, rt. (c) acrylonitrile, 0 °C → rt., then Boc_2O. (d) Raney®-Nickel, ammonia, 3 bar H_2, MeOH, rt.

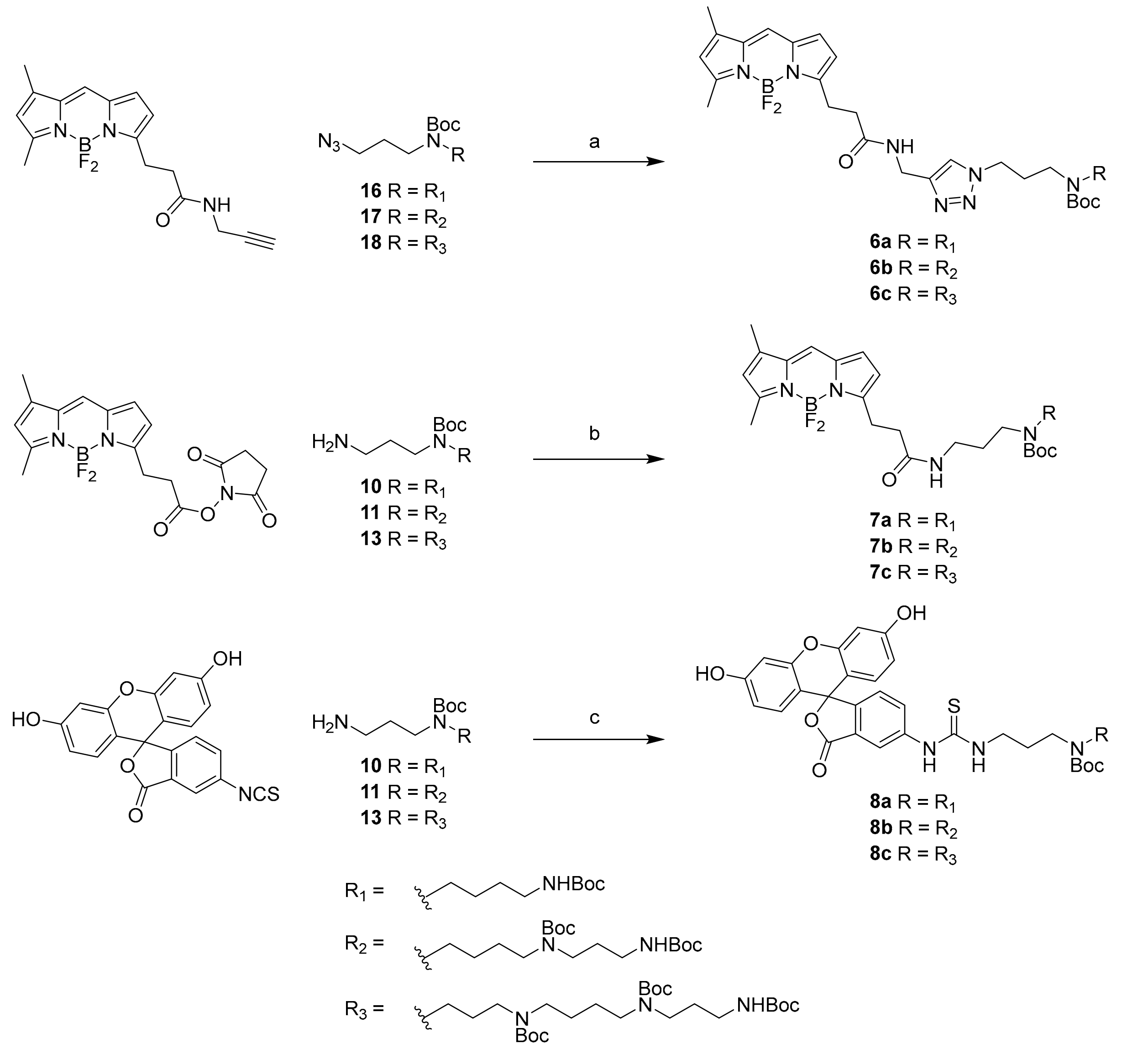

Figure S3. Conjugation of the fluorophore to the relevant protected polyamine. (a) CuBr, DIPEA, DCM, rt. (b) DIPEA, DCM, rt. (c) Et_3N, DCM, rt.

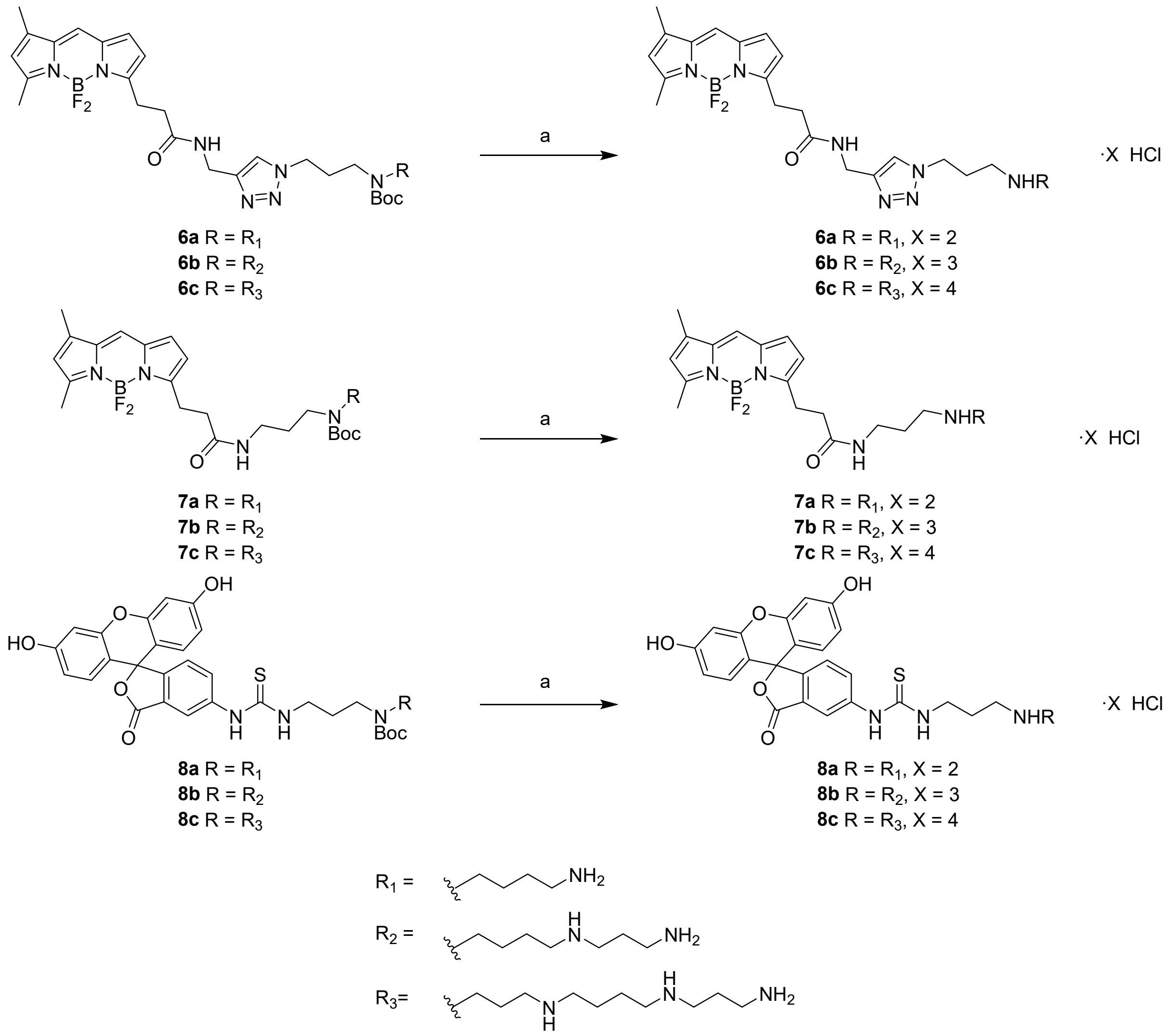

Figure S4. Boc-deprotection of the relevant Boc-protected probe. (a) 4 M HCl/dioxane, rt.

General procedures

<u>*General procedure A for synthesis of Boc-protected BODIPY-FL-A-probes*</u>

The relevant Boc-protected polyamine (1.2 eq) was dissolved in dichloromethane. Diisopropylethylamine (4.0 eq) was added followed by the BODIPY-FL *N*-hydroxysuccinimide ester (1.0 eq). The resulting solution was stirred overnight under argon atmosphere in the absence of light. The solvent was removed under a stream of nitrogen and the crude product was purified by silica chromatography (1/1 PE/EtOAc → PE/EtOAc 1/3).

<u>*General procedure B for synthesis of Boc-protected BODIPY-FL-T probes*</u>

The relevant Boc-protected polyamine (1.2 eq) was dissolved in dichloromethane. Diisopropylethylamine (4.0 eq) was added followed by copper bromide (0.5 eq). Subsequently, the relevant alkyne-BODIPY (1.0 eq) was added. The resulting suspension was stirred overnight under argon atmosphere in the absence of light. Following this, the mixture was washed three times using a saturated sodium bicarbonate solution. The solvent was then removed under a stream of nitrogen gas and the obtained crude product was purified using silica chromatography.

<u>*General procedure C for synthesis of Boc-protected FITC probes*</u>

The relevant Boc-protected polyamine (1.2 eq) was dissolved in dichloromethane. Triethylamine (4.0 eq) was added followed by FITC (1.0 eq). The resulting solution was stirred overnight under argon atmosphere in the absence of light. The solvent was removed under a stream of nitrogen and the crude product was purified by silica chromatography (5% MeOH in DCM + 1% AcOH).

General procedure D for Boc-deprotection towards the final polyamine probes

1.0 mL 4 M HCl/dioxane was added to the relevant Boc-protected polyamine probe (1.0 eq). The resulting solution was left to stand for 2.5 min per present Boc group generating colored precipitate. Following this, the solvent was removed *in vacuo* before dioxane was added. The resulting suspension was concentrated *in vacuo*. This was repeated two more times before the obtained solid was dissolved in water and lyophilised, yielding the final polyamine probe without further purification.

***tert*-Butyl (4-((*tert*-butoxycarbonyl)amino)butyl)(2-cyanoethyl)carbamate (9)**

Putrescine (2.002 g, 1.0 eq) was dissolved in 18 mL of methanol after which the solution was purged *via* nitrogen gas bubbling for 20 minutes and left to stir under nitrogen atmosphere. The solution was cooled to 0 °C and acrylonitrile (1.5 mL, 1.0 eq) was added dropwise over the course of two hours after which the mixture was left to slowly warm to room temperature and further stirred for 24 hours. An additional 20 mL of methanol was added followed by triethylamine (20 mL, 6.3 eq). The mixture was cooled to 0 °C and Boc anhydride (15.039 g, 3.0 eq) was added in portions. The reaction was left to stir overnight at room temperature. The solvent was removed *in vacuo* and the resulting crude oil purified using silica chromatography (4/1 PE/EtOAc → PE/EtOAc 2/1). Pure fractions were pooled and concentrated *in vacuo* resulting in a colorless oil (2.07 g, yield = 26%).

ESI-MS: m/z calcd for $C_{17}H_{32}N_3O_4$ $[M+H]^+$: 342.24; found: 342.15

***tert*-Butyl (3-aminopropyl)(4-((*tert*-butoxycarbonyl)amino)butyl)carbamate (10)**

Nitrile **9** (318.3 mg, 1.0 eq) was added to a glass Parr® vessel and dissolved in 21 mL of 70% aqueous ethanol followed by the addition of 14 mL of a 1 M methanolic sodium hydroxide solution. Three scoops of Raney®-Nickel slurry (50% in water) were added and the formed suspension was put under an atmosphere of hydrogen at 2.3 bars and subsequently shaken overnight. The hydrogen atmosphere was removed and the suspension was filtered over a layer of Celite® 545. The filtrate was concentrated to a minimal volume *in vacuo* after which 75 mL of dichloromethane was added and washed twice using 75 mL of a 0.1 M aqueous sodium hydroxide and once using 75 mL of water. The organic phase was dried using magnesium sulfate, filtered, and concentrated resulting in a yellow oil (0.313 g, yield = 97%) which was used as such in subsequent reactions.

ESI-MS: m/z calcd for $C_{17}H_{36}N_3O_4$ $[M+H]^+$: 346.27; found: 346.90

***tert*-Butyl (4-((3-aminopropyl)(*tert*-butoxycarbonyl)amino)butyl)(3-((*tert*-butoxycarbonyl) amino)propyl)carbamate (11)**

Spermine (1.37 g, 1.3 eq) was dissolved in 40 mL methanol and put under argon atmosphere. Ethyl trifluoroacetate (0.643 mL, 1.0 eq) was dissolved in 20 mL methanol and added dropwise at 0 °C over the course of 30 minutes while stirring vigorously. After addition, the solution was left to warm to room temperature. After three hours of further stirring, Boc anhydride (8.890 g, 7.5 eq) was added in portions. The resulting solution was left to stir overnight under

argon atmosphere. A 2 M aqueous sodium hydroxide solution was added dropwise resulting in the formation of a white precipitate. This suspension was further stirred overnight under argon atmosphere after which the mixture was concentrated to a minimal volume. Water was added to obtain a total volume of 150 mL which was extracted three times using 150 mL chloroform. The organic phases were combined, dried using magnesium sulfate, filtered, and concentrated *in vacuo*. The resulting crude material was purified using silica chromatography (2% MeOH in DCM → 5 % MeOH in DCM → 5 % MeOH in DCM + 1% Et_3N → 10% MeOH in DCM + 1% Et_3N) resulting in a colorless oil (1.639 g, yield = 60%).

ESI-MS: m/z calcd for $C_{25}H_{51}N_4O_6$ $[M+H]^+$: 503.38; found: 503.35

***tert*-Butyl (4-((*tert*-butoxycarbonyl)(3-((*tert*-butoxycarbonyl)(2-cyanoethyl)amino)propyl)amino)butyl)(3-((*tert*-butoxycarbonyl)amino)propyl)carbamate (12)**

Acrylonitrile (0.146 mL, 1.0 eq) was added dropwise to a solution of amine **11** (1.122 g, 1.0 eq) in 6 mL methanol, cooled to 0 °C. The resulting solution was stirred overnight under argon atmosphere after which triethylamine (1.24 mL, 4.0 eq) was added followed by the addition of Boc anhydride (1.43 g, 2.9 eq) in small portions. The solution was stirred overnight before being concentrated *in vacuo*. The crude mixture was purified using silica column chromatography (2/1 PE/EtOAC → 1/2 PE/EtOAc) resulting in a colorless oil (0.974, yield = 67%).

ESI-MS: m/z calcd for $C_{33}H_{62}N_5O_8$ $[M+H]^+$: 656.46; found: 656.45

***tert*-Butyl (4-((3-((3-aminopropyl)(*tert*-butoxycarbonyl)amino)propyl)(*tert*-butoxycarbonyl)amino)butyl)(3-((*tert*-butoxycarbonyl)amino)propyl)carbamate (13)**

Nitrile **12** (0.974 g, 1.0 eq) was dissolved in 10 mL of a 2 M methanolic ammonia solution followed by the addition of three scoops of a Raney®-Nickel slurry (50% in water). The resulting suspension was shaken overnight in a glass Parr® vessel under a hydrogen atmosphere of 3 bar. After removal of the hydrogen atmosphere solids were filtered off over Celite® 545. The filtrate was concentrated *in vacuo* and the obtained crude purified using silica chromatography (5% MeOH in DCM + 1% Et_3N). Clean fractions were pooled resulting in a colorless oil (0.616 g, yield = 63%).

ESI-MS: m/z calcd for $C_{33}H_{66}N_5O_8$ $[M+H]^+$: 660.49; found: 660.50

***tert*-Butyl (4-((*tert*-butoxycarbonyl)amino)butyl)(3-(4-((3-(5,5-difluoro-7,9-dimethyl-5*H*-4λ⁴, 5λ⁴-dipyrrolo[1,2-c:2',1'-*f*][1,3,2]diazaborinin-3-yl)propanamido)methyl)-1*H*-1,2,3-triazol-1-yl)propyl)carbamate (6a)**

Prepared according to general procedure B using azide **16** (10.76 mg) as starting material. The crude was purified by silica chromatography (3% MeOH in DCM → 5% MeOH in DCM) resulting in a red solid (12.28 mg, yield = 61%).

^{1}H-NMR (600 MHz, DMSO-d6): δ = 8.42 8.37 (m, 1H), 7.88 (s, 1H), 7.64 (s, 1H), 7.03 (d, 1H, J = 3.76 Hz), 6.77 – 6.72 (m, 1H), 6.29 (d, 1H, J = 3.80 Hz), 6.26 (s, 1H), 4.29 – 4.22 (m, 4H), 3.13 – 3.01 (m, 7H), 2.89 – 2.82 (m, 2 H), 2.51 (s, 2H), 2.42 (s, 3H), 2.21 (s, 3H), 1.98 – 1.91 (m, 2H), 1.41 – 1.22 (m, 19H), 1.13 (t, 2H, J = 7.27) ^{13}C-NMR (150 MHz, DMSO-d6)(rotamers): δ = 170.78, 159.20, 157.72, 156.85, 155.63, 155.15, 154.76, 154.48, 144.84, 144.13, 134.46, 132.96, 128.89, 125.33, 122.81, 120.30, 116.62, 78.52, 77.37, 47.22, 46.35, 46.02, 45.74, 45.70, 43.84, 43.73, 34.35,

34.23, 33.60, 29.03, 28.74, 28.26, 28.01, 26.88, 25.54, 25.12, 23.92, 23.29, 18.06, 16.72, 14.51, 10.99, 8.61.

ESI-MS: m/z calcd for $C_{34}H_{52}BF_2N_8O_5$ [M+H]$^+$: 701.41; found: 701.40

***tert*-Butyl (4-((*tert*-butoxycarbonyl)(3-((*tert*-butoxycarbonyl)amino)propyl)amino)butyl)(3-(4-((3-(5,5-difluoro-7,9-dimethyl-5*H*-4λ^4,5λ^4-dipyrrolo[1,2-*c*:2',1'-*f*][1,3,2]diazaborinin-3-yl)propanamido)methyl)-1*H*-1,2,3-triazol-1-yl)propyl)carbamate (6b)**
Prepared according to general procedure B using azide **17** (10.16 mg) as starting material. The crude was purified by silica chromatography (3% MeOH in DCM → 5% MeOH in DCM) resulting in a red solid (9.90 mg, yield = 65%).

^{1}H-NMR (600 MHz, DMSO-d6): δ = 8.36 (s, 1H), 7.88 (s, 1H), 7.60 (s, 1H), 7.00 (d, 1H, J = 3.82 Hz), 6.67 (br s, 1H), 6.26 (d, 1H, J = 3.90 Hz), 6.23 (s, 1H), 4.29 – 4.17 (m, 4H), 3.11 – 2.96 (m, 10H), 2.83 – 2.77 (m, 2H), 2.39 (s, 3H), 2.18 (s, 3H), 1.96 – 1.87 (m, 2H), 1.47 (br s, 2H), 1.37 – 1.19 (br, s 33H) ^{13}C-NMR (150 MHz, DMSO-d6)(rotamers): δ = 170.81, 159.20, 157.66, 155.55, 154.65, 144.13, 134.44, 132.93, 128.86, 125.30, 123.13, 120.29, 116.59, 78.52, 78.29, 77.46, 47.23, 46.47, 45.94, 44.40, 44.18, 43.84, 37.58, 34.26, 33.62, 28.90, 28.22, 28.01, 27.97, 25.57, 25.08, 23.90, 14.48, 10.97.

ESI-MS: m/z calcd for $C_{42}H_{67}BF_2N_9O_7$ [M+H]$^+$: 858.52; found: 858.50

***tert*-Butyl (4-((*tert*-butoxycarbonyl)(3-((*tert*-butoxycarbonyl)amino)propyl)amino)butyl)(3-((*tert*-butoxycarbonyl)(3-(4-((3-(5,5-difluoro-7,9-dimethyl-5*H*-4λ^4,5λ^4-dipyrrolo[1,2-*c*:2',1'-*f*][1,3,2]diazaborinin-3-yl)propanamido)methyl)-1*H*-1,2,3-triazol-1-yl)propyl)carbamate (6c)**
Prepared according to general procedure B using azide **18** (10.12 mg) as starting material. The crude was purified by silica chromatography (3% MeOH in DCM → 5% MeOH in DCM) resulting in a red solid (4.12 mg, yield = 45%).

^{1}H-NMR (600 MHz, DMSO-d6): δ = 8.42 (s, 1H), 7.94 (s, 1H), 7.67 (s, 1H), 7.06 (d, 1H, J = 3.84), 6.72 (br s, 1H), 6.32 (d, 1H, J = 3.88), 6.29 (s, 1H), 4.34 – 4.25 (m, 3H), 3.18 – 3.01 (m, 16H), 2.90 – 2.84 (m, 2H), 2.45 (s, 3H), 2.25 (s, 3H), 2.03 – 1.94 (m, 2H), 1.62 (br s, 2H), 1.54 (br s, 2H), 1.42 – 1.31 (m, 41H) ^{13}C-NMR (150 MHz, DMSO-d6)(rotamers): δ = 170.79, 159.20, 157.67, 155.55, 154.64, 154.55, 144.12, 134.44, 132.94, 128.87, 125.30, 123.10, 120.29, 116.59, 78.60, 78.30, 78.27, 77.45, 47.20, 46.36, 45.93, 45.77, 44.36, 44.14 ,43.82, 37.57, 34.26, 33.62, 28.88, 25.65, 25.11, 23.90, 14.48, 10.97, 8.62.

ESI-MS: m/z calcd for $C_{50}H_{82}BF_2N_{10}O_9$ [M+H]$^+$: 1015.63; found: 1015.65

***tert*-Butyl (4-((*tert*-butoxycarbonyl)amino)butyl)(3-(3-(5,5-difluoro-7,9-dimethyl-5*H*-4λ^4,5λ^4-dipyrrolo[1,2-*c*:2',1'-*f*][1,3,2]diazaborinin-3-yl)propanamido)propyl)carbamate (7a)**
Prepared according to general procedure A using amine **10** (7.23 mg) as starting material resulting in a red solid (9.2 mg, yield = 80%).

^{1}H-NMR (600 MHz, $CDCl_3$): δ = 7.08 (s, 1H), 6.87 (d, 1H, *J* = 3.2 Hz), 6.29 (d, 1H, J = 3.8 Hz), 6.10 (s, 1H), 3.32 – 3.26 (m, 2H), 3.19 (q, 3H, J = 6.2), 3.17 – 3.08 (m, 6H), 2.63 (t, 2H, J = 7.47), 2.56 (s, 3H), 2.25 (s, 3H), 1.62 (quint, 2H, J = 6.4), 1.55 – 1.47 (m, 1H), 1.43 (s, 19H), 1.36 – 1.30 (m, 1H) ^{13}C-NMR (150 MHz, $CDCl_3$)(rotamers): δ = 171.84, 156.14, 143.69, 133.50, 128.40, 123.88, 120.39,

117.47, 79.76, 79.28, 46.82, 45.78, 44.86, 43.78, 40.27, 37.23, 36.16, 35.96, 32.06, 31.57, 30.33, 29.84, 29.50, 29.40, 28.56, 27.98, 27.54, 25.90, 24.90, 22.83, 15.05, 14.26, 11.44, 8.65.

ESI-MS: m/z calcd for $C_{31}H_{48}BF_2N_5O_5$ [M+H]$^+$: 620.38; found: 620.40

***tert*-Butyl (4-((*tert*-butoxycarbonyl)(3-(3-(5,5-difluoro-7,9-dimethyl-5*H*-4λ⁴,5λ⁴-dipyrrolo[1,2-*c*:2',1'-*f*][1,3,2]diazaborinin-3-yl)propanamido)propyl)amino)butyl)(3-((*tert*-butoxycarbonyl)amino)propyl)carbamate (7b)**

Prepared according to general procedure A using amine **11** (7.08 mg) as starting material resulting in a red solid (10.68 mg, yield = 76%).

^{1}H-NMR (600 MHz, $CDCl_3$): δ = 7.07 (s, 1H), 6.87 (d, 1H, J = 2.3 Hz), 6.29 (d, 1H, J = 3.6 Hz), 6.10 (s, 1H), 3.34 – 3.02 (m, 14H), 2.62 (t, 2H, J = 7.44), 2.55 (s, 3H), 2.24 (s, 1H), 1.67 – 1.58 (m, 4H), 1.51 – 1.36 (m, 30H), 1.36 – 1.29 (m, 1H) ^{13}C-NMR (150 MHz, $CDCl_3$)(rotamers): δ = 171.85, 156.27, 155.57, 143.62, 135.17, 133.48, 128.38, 123.86, 120.38, 117.42, 79.75, 46.95, 46.35, 45.76, 44.83, 44.30, 43.76, 37.81, 37.51, 36.11, 35.94, 32.06, 31.57, 30.32, 29.83, 29.49, 29.40, 29.02, 28.58, 27.96, 26.21, 25.54, 24.89, 22.82, 15.05, 14.25, 11.44, 8.64.

ESI-MS: m/z calcd for $C_{39}H_{64}BF_2N_6O_7$ [M+H]$^+$: 777.49; found: 777.50

***tert*-Butyl (4-((*tert*-butoxycarbonyl)(3-((*tert*-butoxycarbonyl)(3-(3-(5,5-difluoro-7,9-dimethyl-5*H*-4λ⁴,5λ⁴-dipyrrolo[1,2-c:2',1'-f][1,3,2]diazaborinin-3-yl)propanamido)propyl)amino)propyl)amino)butyl)(3-((*tert*-butoxycarbonyl)amino)propyl)carbamate (7c)**

Prepared according to general procedure A using amine **18** (7.21 mg) as starting material resulting in a red solid (17.09 mg, yield = 99%).

^{1}H-NMR (600 MHz, $CDCl_3$): δ = 7.07 (s, 1H), 6.87 (d, 1H, J = 3.5 Hz), 6.28 (d, 1H, J = 4.00 Hz), 6.10 (s, 1H), 3.32 – 3.03 (m, 18H), 2.61 (t, 2H, J = 7.58 Hz), 2.54 (s, 3H), 2.24 (s, 3H), 1.75 – 1.59 (m, 6H), 1.50 – 1.37 (m, 40H) ^{13}C-NMR (150 MHz, $CDCl_3$)(rotamers): δ = 171.80, 170.82, 160.02, 158.24, 157.64, 156.23, 155.57, 143.63, 137.26, 135.15, 133.48, 131.04, 129.61, 128.95, 128.39, 124.58, 124.09, 123.87, 120.37, 119.21, 117.42, 99.49, 79.82, 79.62, 79.51, 79.23, 79.01, 66.32, 58.00, 54.50, 46.95, 46.64, 46.48, 44.96, 44.30, 43.72, 39.49, 39.19, 39.03, 38.97, 37.80, 37.47, 37.22, 37.15, 36.05, 35.94, 32.05, 31.56, 28.57, 28.10, 27.89, 26.13, 25.66, 24.99, 24.87, 22.81, 17.75, 17.56, 17.37, 15.04, 14.24, 11.42, 10.15, 8.10.

ESI-MS: m/z calcd for $C_{47}H_{79}BF_2N_7O_9$ [M+H]$^+$: 934.60; found: 934.60

***tert*-Butyl (4-((*tert*-butoxycarbonyl)amino)butyl)(3-(3-(3',6'-dihydroxy-3-oxo-3*H*-spiro[isobenzofuran-1,9'-xanthen]-5-yl)thioureido)propyl)carbamate (8a)**

Prepared according to general procedure C using amine **14** (7.17 mg) as starting material resulting in a yellow solid (17.09 mg, yield = 99%).

^{1}H-NMR (600 MHz, CD_3OD): δ = 8.12 (s, 1H), 7.77 (d, 1H, J = 7.90 Hz), 7.18 (d, 1H, J = 8.18 Hz), 6.82 – 6.65 (m, 4H), 6.59 (d, 2H, J = 7.50 Hz), 3.63 (s, 2H), 3.23 (t, 2H, J = 7.32 Hz), 3.05 (t, 2H, J = 6.87), 1.98 – 1.81 (m, 2H), 1.62 – 1.53 (m, 2H), 1.51 – 1.37 (m, 23H) ^{13}C-NMR (150 MHz, CD_3OD)(rotamers): δ = 210.11, 182.96, 182.61, 170.76, 162.16, 158.58, 157.83, 157.39, 154.75, 142.49, 142.20, 131.78, 130.68, 130.19, 129.37, 126.29, 120.71, 114.22, 112.04, 103.49, 81.08, 79.87,

48.09, 47.95, 46.23, 45.18, 43.50, 42.72, 42.36, 40.98, 30.76, 30.67, 29.35, 28.81, 28.79, 28.57, 28.37, 27.02, 26.52, 24.22.

ESI-MS: m/z calcd for $C_{38}H_{47}N_4O_9S$ [M+H]$^+$: 735.31; found: 735.35

***tert*-Butyl (4-((*tert*-butoxycarbonyl)(3-(3-(3',6'-dihydroxy-3-oxo-3*H*-spiro[isobenzofuran-1,9'-xanthen]-5-yl)thioureido)propyl)amino)butyl)(3-((*tert*-butoxycarbonyl)amino)propyl)carbamate (8b)**

Prepared according to general procedure C using amine **11** (7.43 mg) as starting material resulting in a yellow solid (17.09 mg, yield = 99%).

^{1}H-NMR (600 MHz, CD_3OD): δ = 8.09 (s, 1H), 7.74 (d, 1H, J = 7.71 Hz), 7.16 (d, 1H, J = 8.21 Hz), 6.74 – 6.65 (m, 4H), 6.54 (dd, 1H, J = 8.62 Hz, 1.94 Hz), 3.62 (s, 2H), 3.27 – 3.18 (m, 6H), 3.02 (t, J = 6.82 Hz), 1.95 – 1.80 (m, 2H), 1.68 (s, 2H), 1.58 – 1.37 (m, 33H) ^{13}C-NMR (150 MHz, CD_3OD)(rotamers): δ = 210.09, 182.94, 182.61, 171.12, 161.74, 158.43, 157.77, 157.38, 154.30, 149.56, 142.30, 142.06, 131.94, 130.39, 129.45, 125.96, 120.30, 113.81, 111.58, 103.53, 81.07, 80.99, 79.97, 48.28, 47.91, 47.78, 46.26, 46.11, 45.66, 45.29, 43.51, 42.72, 38.93, 38.85, 30.77, 30.69, 30.26, 29.47, 28.82, 27.27, 27.14, 26.67, 26.53.

ESI-MS: m/z calcd for $C_{46}H_{62}N_5O_{11}S$ [M+H]$^+$: 892.42; found: 892.45

***tert*-Butyl (4-((*tert*-butoxycarbonyl)(3-((*tert*-butoxycarbonyl)(3-(3-(3',6'-dihydroxy-3-oxo-3*H*-spiro[isobenzofuran-1,9'-xanthen]-5-yl)thioureido)propyl)amino)propyl)amino)butyl)(3-((*tert* butoxycarbonyl)amino)propyl)carbamate (8c)**

Prepared according to general procedure C using amine **18** (7.18 mg) as starting material resulting in a yellow solid (19.08 mg, yield = 99%).

^{1}H-NMR (600 MHz, CD3OD): δ = 8.08 (s, 1H), 7.74 (d, 1H, J = 7.84 Hz), 7.16 (d, 1H, J = 8.22), 6.75 – 6.64 (m, 4H), 6.54 (dd, 2H, J = 8.70 Hz, 2.10 Hz), 3.63 (s, 2H), 3.27 – 3.12 (m, 12H), 3.02 (t, 2H, J = 6.69 Hz), 1.98 – 1.83 (m, 2H), 1.80 (q, 2H, J = 14.04 Hz), 1.67 (s, 2H), 1.55 – 1.38 (m, 40H) ^{13}C-NMR (150 MHz, CD_3OD)(rotamers): δ = 210.11, 182.89, 182.59, 171.18, 169.33, 169.30, 162.33, 158.41, 157.30, 154.51, 148.80, 142.24, 142.06, 133.57, 132.41, 132.36, 131.66, 130.49, 129.87, 126.18, 120.44, 114.16, 111.80, 103.56, 81.17, 80.96, 79.95, 69.53, 48.26, 47.87, 47.75, 46.24, 46.05, 45.62, 45.24, 43.47, 42.74, 38.92, 38.83, 38.41, 34.80, 33.30, 33.06, 32.41, 30.75, 30.68, 30.61, 30.46, 30.29, 30.22, 30.13, 29.47, 28.84, 28.81, 27.54, 27.23, 27.05, 26.63, 23.72, 23.67, 20.97, 14.73, 14.44, 14.41, 9.20.

ESI-MS: m/z calcd for $C_{54}H_{77}N_6O_{13}S$ [M+H]$^+$: 1049.53; found: 1049.55

***N*-((1-(3-((4-Aminobutyl)amino)propyl)-1*H*-1,2,3-triazol-4-yl)methyl)-3-(5,5-difluoro-7,9-dimethyl-5*H*-4λ^4,5λ^4-dipyrrolo[1,2-*c*:2',1'-*f*][1,3,2]diazaborinin-3-yl)propenamide dihydrochloride (2a)**

Prepared according to general procedure D using Boc-protected probe **6a** (5.82 mg) as starting material. Obtained without purification as a red solid (4.65 mg, yield = 98%).

HRMS: m/z calcd for $C_{24}H_{36}BF_2N_8O$ [M-H2Cl]$^+$: 501.3073; found: 501.3074

N-((1-(3-((4-((3-Aminopropyl)amino)butyl)amino)propyl)-1H-1,2,3-triazol-4-yl)methyl)-3-(5,5-difluoro-7,9-dimethyl-5H-4λ⁴,5λ⁴-dipyrrolo[1,2-c:2',1'-f][1,3,2]diazaborinin-3-yl)propenamide trihydrochloride (2b)
Prepared according to general procedure D using Boc-protected probe **6b** (7.06 mg) as starting material. Obtained without purification as a red solid (5.15 mg, yield = 94%).

HRMS: m/z calcd for $C_{27}H_{43}BF_2N_9O$ [M-2H3Cl]$^+$: 558.3651; found: 558.3652

N-((1-(3-((3-((4-((3-Aminopropyl)amino)butyl)amino)propyl)amino)propyl)-1H-1,2,3-triazol-4-yl)methyl)-3-(5,5-difluoro-7,9-dimethyl-5H-4λ⁴,5λ⁴-dipyrrolo[1,2-c:2',1'-f][1,3,2]diazaborinin-3-yl)propenamide tetrahydrochloride (2c)
Prepared according to general procedure D using Boc-protected probe **6c** (5.29 mg) as starting material. Obtained without purification as a red solid (3.2 mg, yield = 81%).

HRMS: m/z calcd for $C_{30}H_{50}BF_2N_{10}O$ [M-3H4Cl]$^+$:615.4230; found: 615.4225

N-(3-((4-Aminobutyl)amino)propyl)-3-(5,5-difluoro-7,9-dimethyl-5H-4λ⁴,5λ⁴-dipyrrolo[1,2-c:2',1'-f][1,3,2]diazaborinin-3-yl)propenamide dihydrochloride (3a)
Prepared according to general procedure D using Boc-protected probe **7a** (3.54 mg) as starting material. Obtained without purification as a red solid (3.84 mg, yield = 99%).

HRMS: m/z calcd for $C_{21}H_{33}BF_2N_5O$ [M-H2Cl]$^+$: 420.2746; found: 420.2747

N-(3-((4-((3-Aminopropyl)amino)butyl)amino)propyl)-3-(5,5-difluoro-7,9-dimethyl-5H-4λ⁴,5λ⁴-dipyrrolo[1,2-c:2',1'-f][1,3,2]diazaborinin-3-yl)propenamide trihydrochloride (3b)
Prepared according to general procedure D using Boc-protected probe **7b** (3.22 mg) as starting material. Obtained without purification as a red solid (2.41 mg, yield = 99%).

HRMS: m/z calcd for $C_{24}H_{40}BF_2N_6O$ [M-2H3Cl]$^+$: 477.3324; found: 477.3322

N-(3-((3-((4-((3-Aminopropyl)amino)butyl)amino)propyl)amino)propyl)-3-(5,5-difluoro-7,9-dimethyl-5H-4λ⁴,5λ⁴-dipyrrolo[1,2-c:2',1'-f][1,3,2]diazaborinin-3-yl)propenamide tetrahydrochloride (3c)
Prepared according to general procedure D using Boc-protected probe **7c** (4.01 mg) as starting material. Obtained without purification as a red solid (2.39 mg, yield = 97%).

HRMS: m/z calcd for $C_{27}H_{47}BF_2N_7O$ [M-3H4Cl]$^+$: 534.3903; found: 534.3909

1-(3-((4-Aminobutyl)amino)propyl)-3-(3',6'-dihydroxy-3-oxo-3H-spiro[isobenzofuran-1,9'-xanthen]-5-yl)thiourea dihydrochloride (4a)
Prepared according to general procedure D using Boc-protected probe **8a** (3.61 mg) as starting material. Obtained without purification as a yellow solid (2.92 mg, yield = 98%).

HRMS: m/z calcd for $C_{28}H_{31}N_4O_5S$ [M-H2Cl]$^+$: 535.2015; found: 535.2012

1-(3-((4-((3-Aminopropyl)amino)butyl)amino)propyl)-3-(3',6'-dihydroxy-3-oxo-3H-spiro[isobenzofuran-1,9'-xanthen]-5-yl)thiourea trihydrochloride (4b)
Prepared according to general procedure D using Boc-protected probe **8b** (4.80 mg) as starting material. Obtained without purification as a yellow solid (3.71 mg, yield = 98%).

HRMS: m/z calcd for $C_{31}H_{38}N_5O_5S$ [M-2H3Cl]$^+$: 592.2593; found: 592.2598

1-(3-((3-((4-((3-Aminopropyl)amino)butyl)amino)propyl)amino)propyl)-3-(3',6'-dihydroxy-3-oxo-3*H*-spiro[isobenzofuran-1,9'-xanthen]-5-yl)thiourea tetrahydrochloride (4c)
Prepared according to general procedure D using Boc-protected probe **8c** (4.95 mg) as starting material. Obtained without purification as a yellow solid (3.6 mg, yield = 96%).

HRMS: m/z calcd for $C_{34}H_{45}N_6O_5S$ [M-3H4Cl]+: 649.3172; found: 649.3174

^{1}H-NMR and ^{13}C-NMR data

Boc-protected BODIPY-FL-T **6a**
^{1}H-NMR

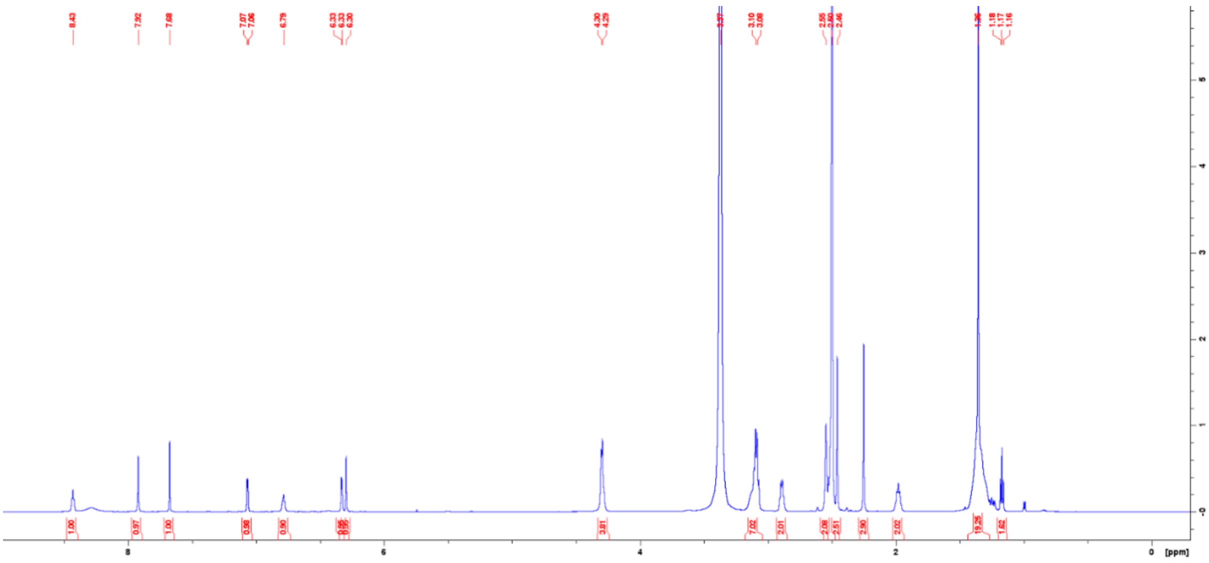

^{13}C-NMR

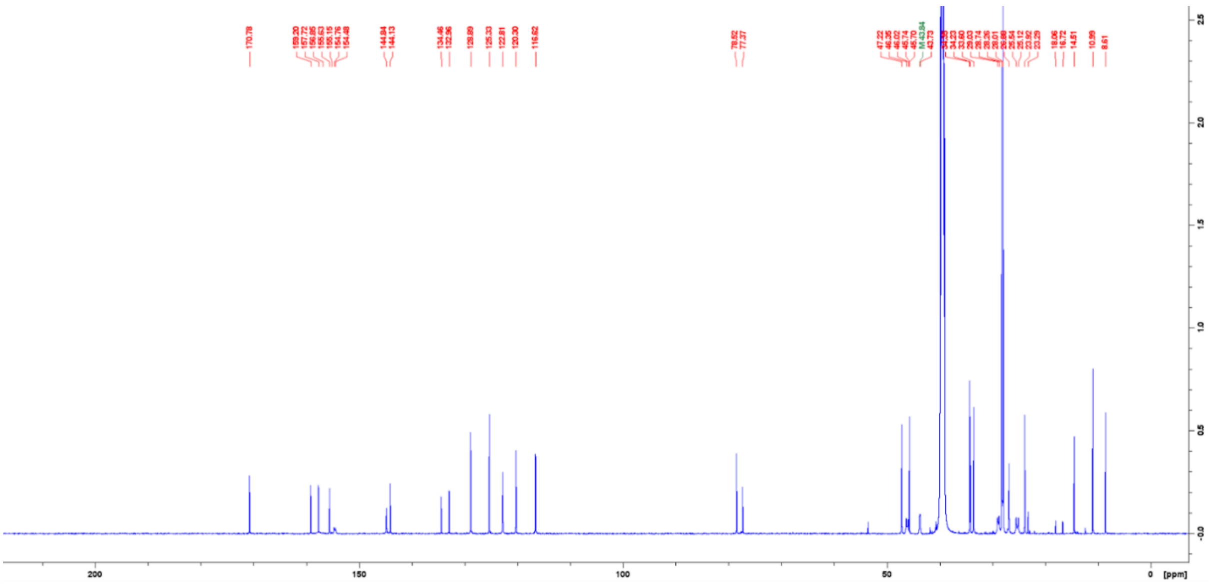

Boc-protected BODIPY-FL-T **6b**
^{1}H-NMR

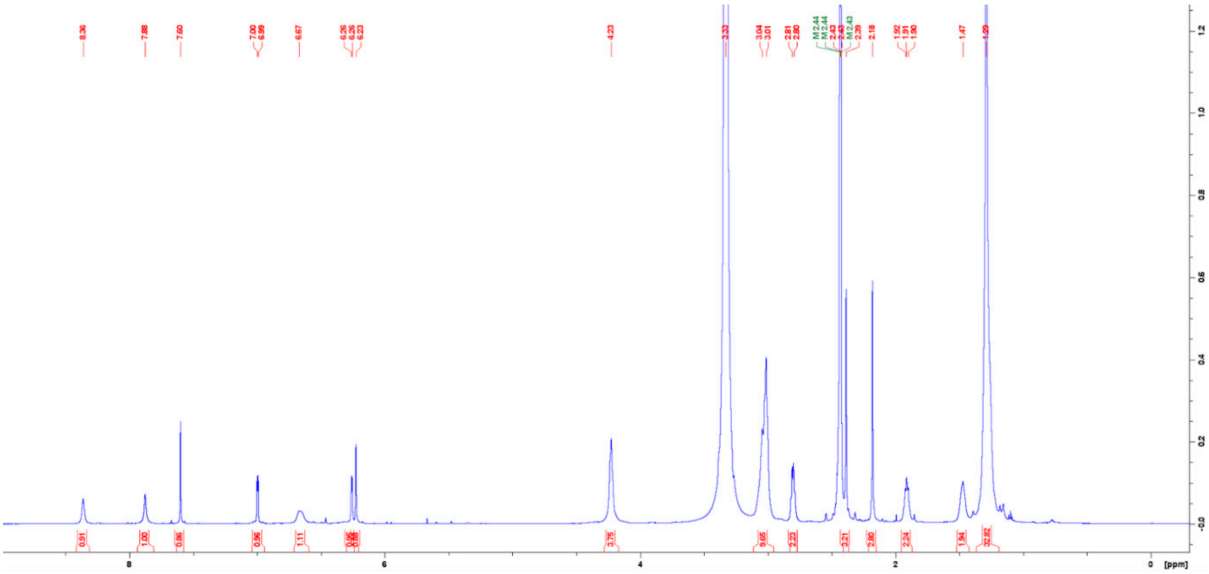

^{13}C-NMR

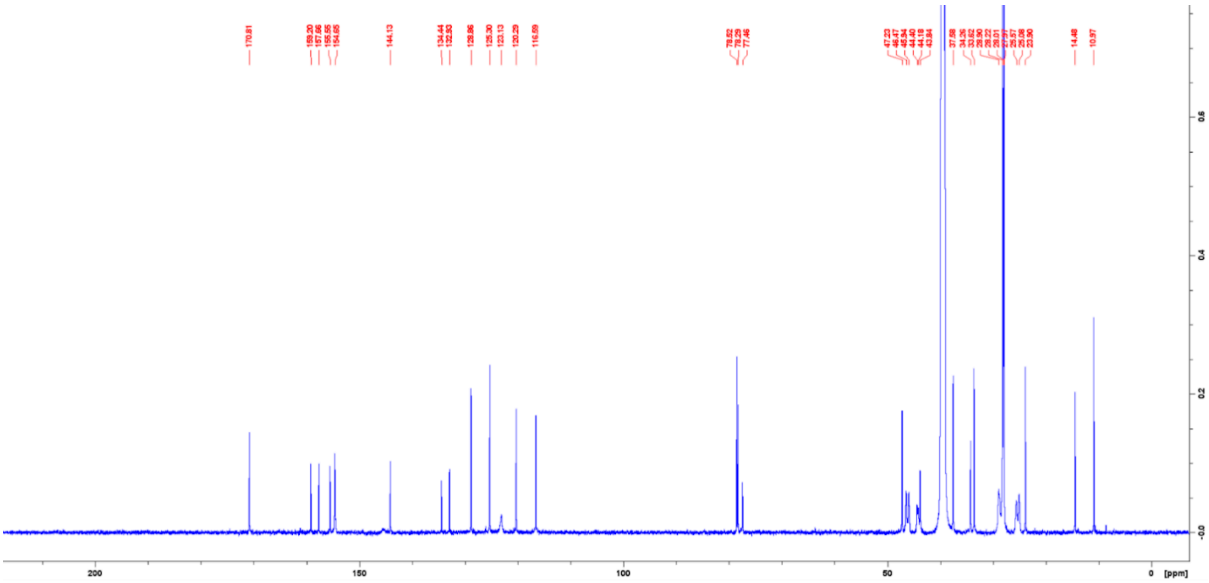

Boc-protected BODIPY-FL-T **6c**
^{1}H-NMR

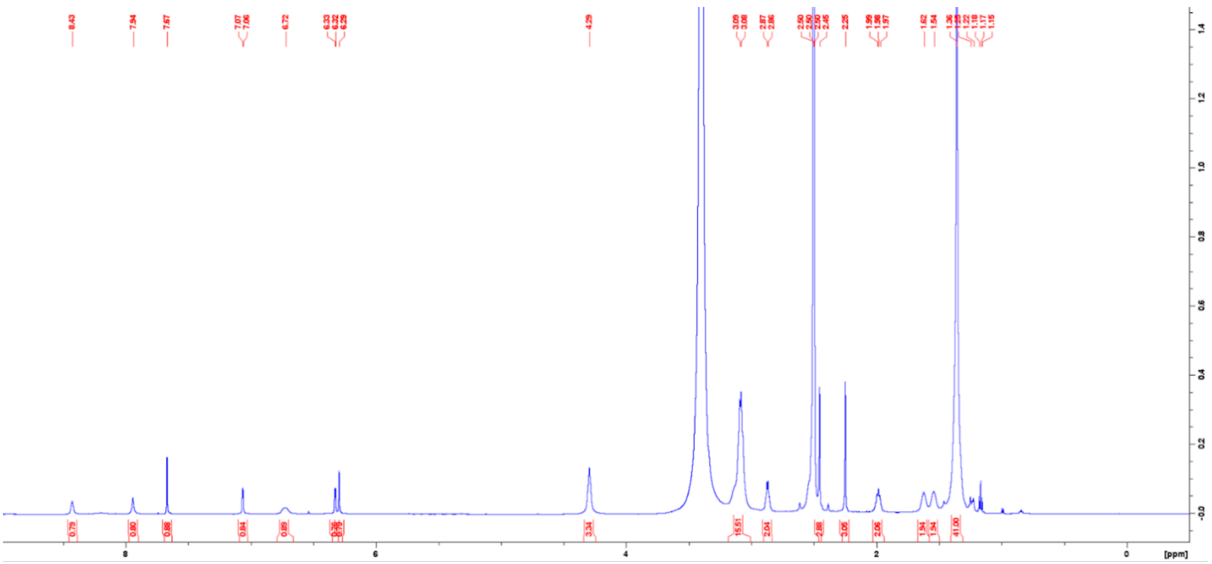

^{13}C-NMR

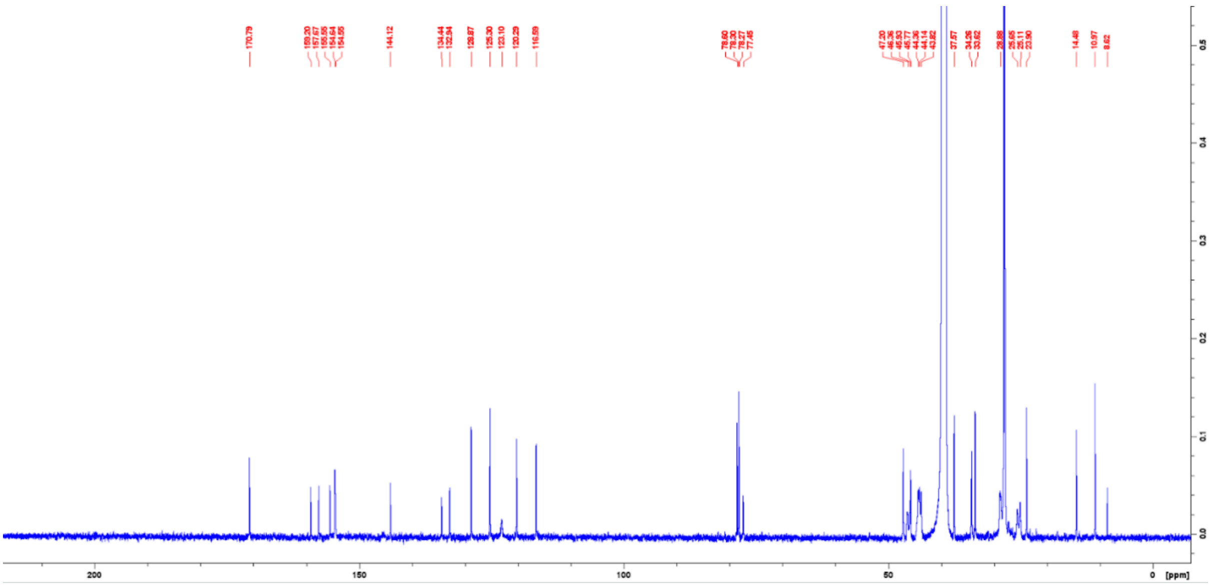

Boc-protected BODIPY-FL-A **7a**
^{1}H-NMR

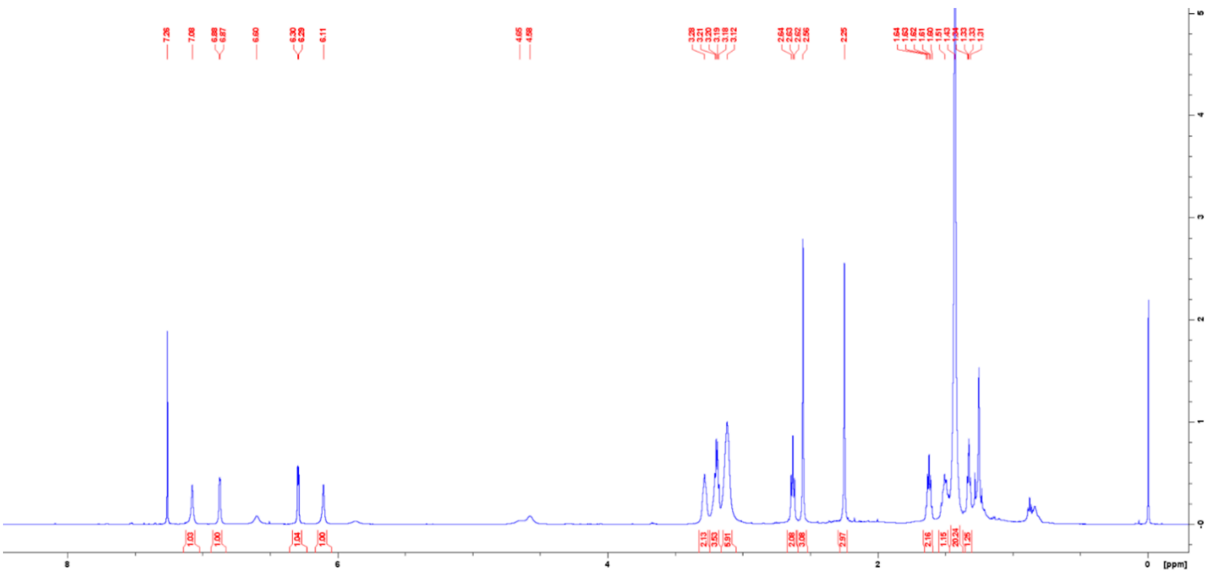

^{13}C-NMR

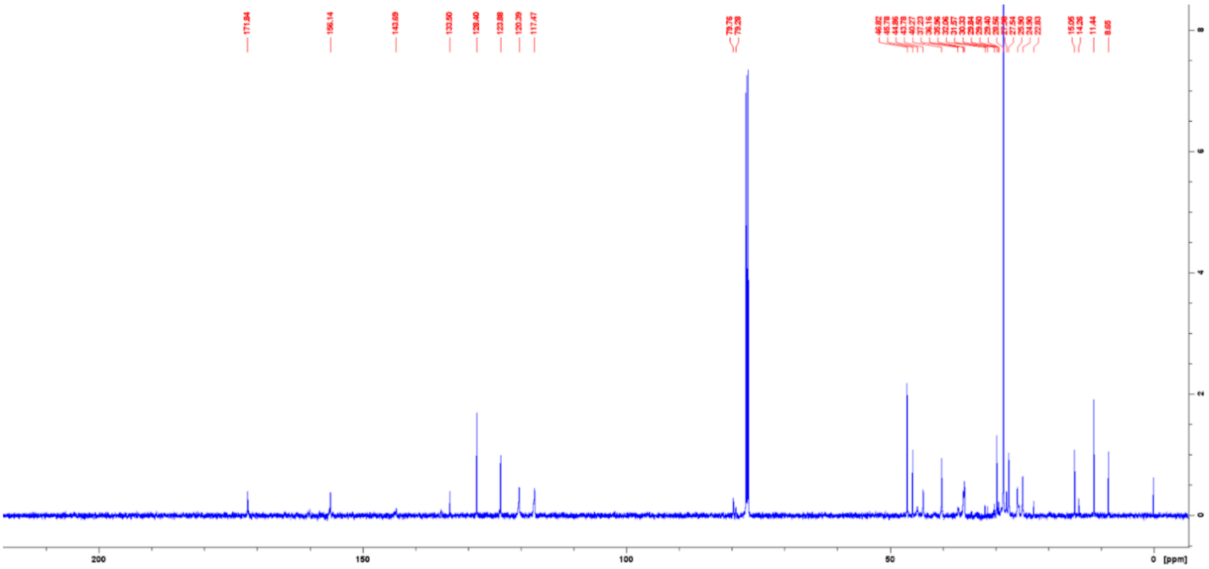

Boc-protected BODIPY-FL-A **7b**

^{1}H-NMR

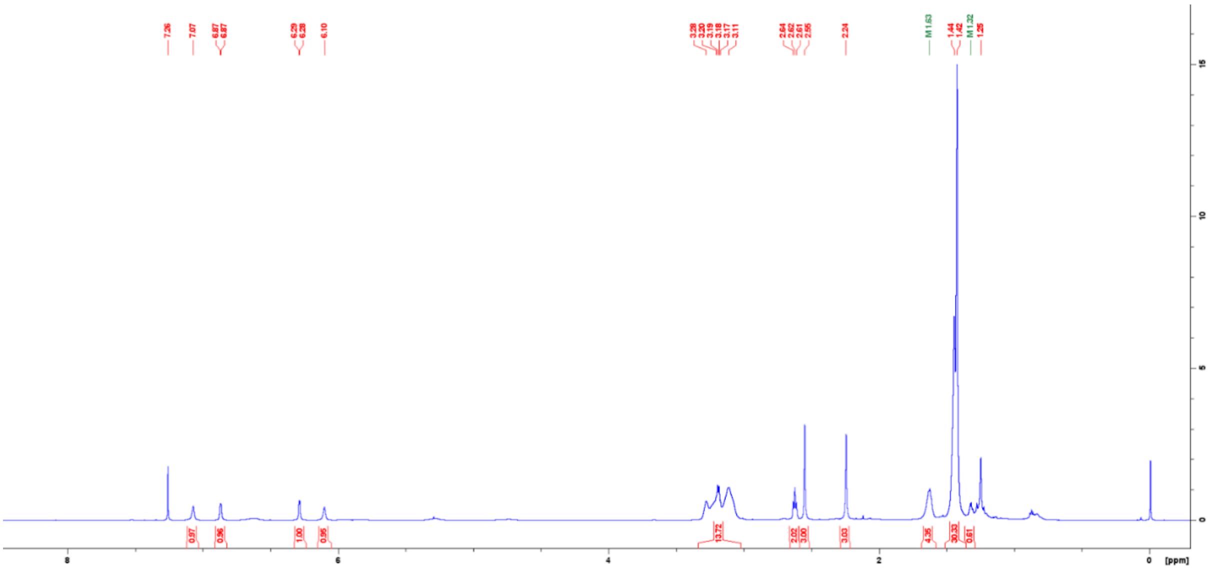

^{13}C-NMR

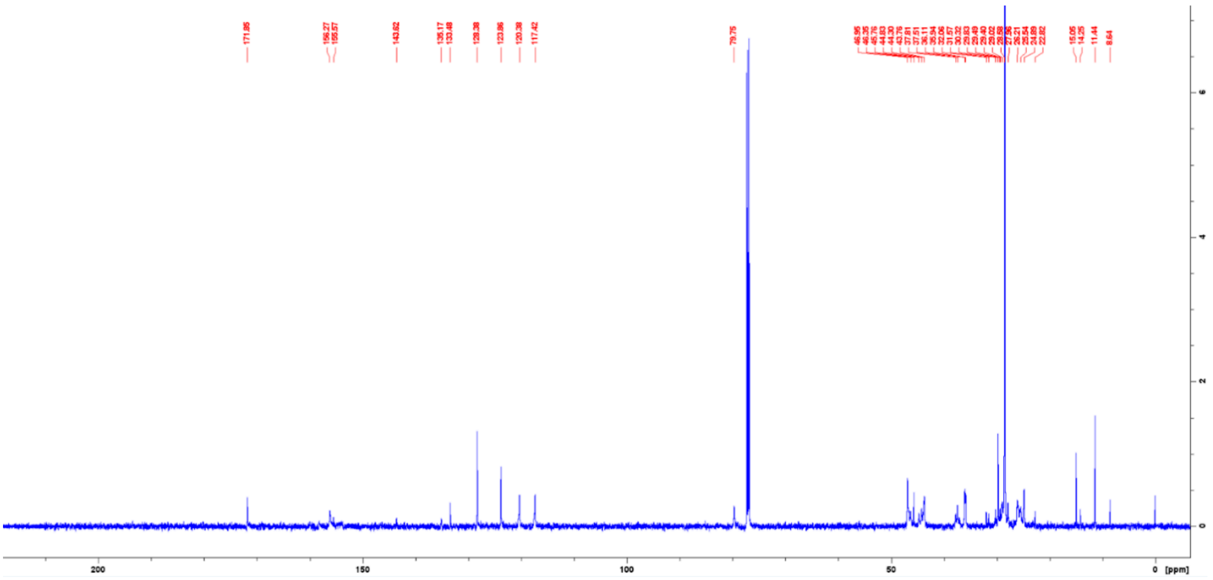

Boc-protected BODIPY-FL-A **7c**

^{1}H-NMR

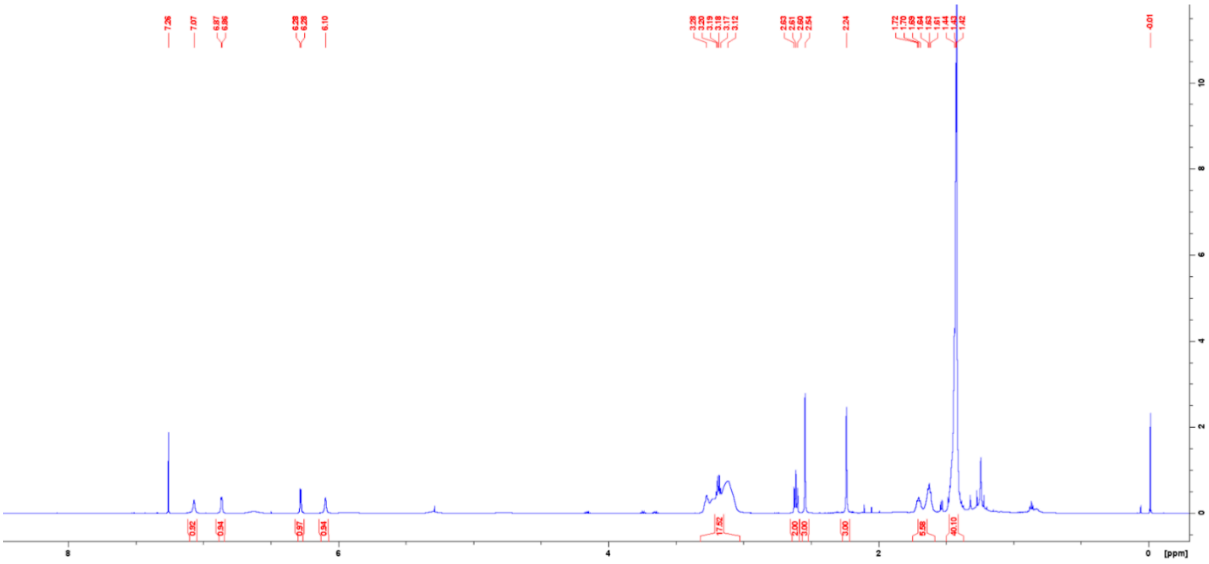

^{13}C-NMR

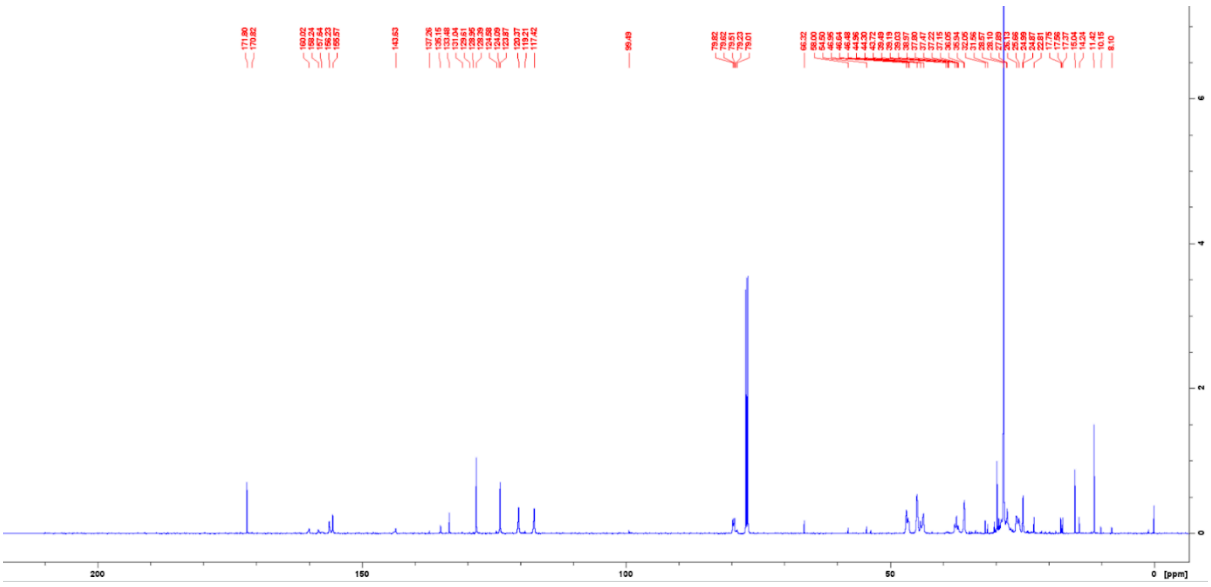

Boc-protected FITC probe **8a**

^{1}H-NMR

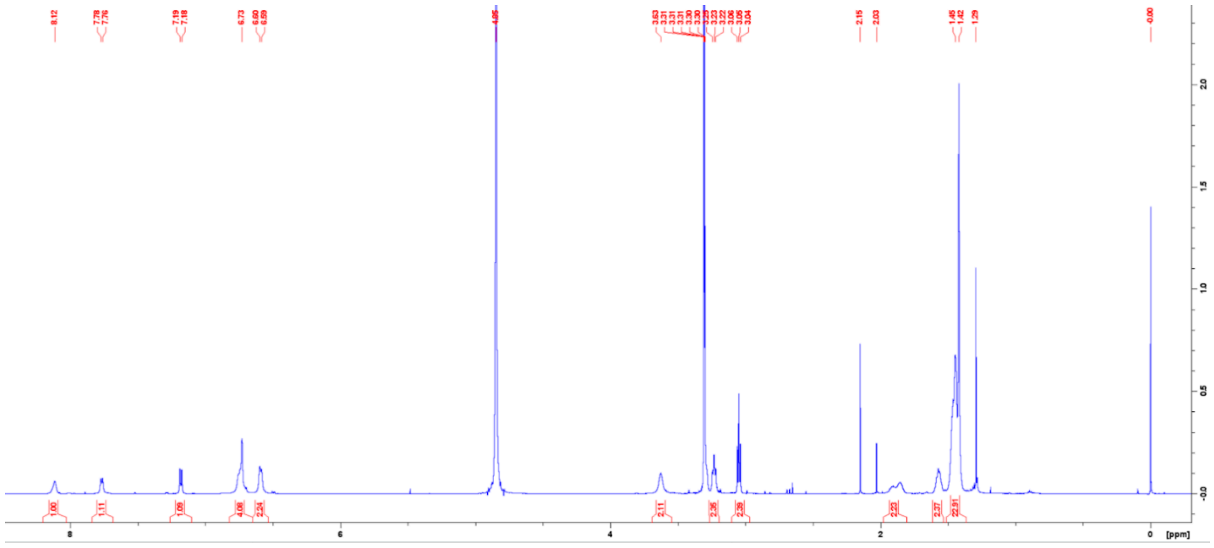

^{13}C-NMR

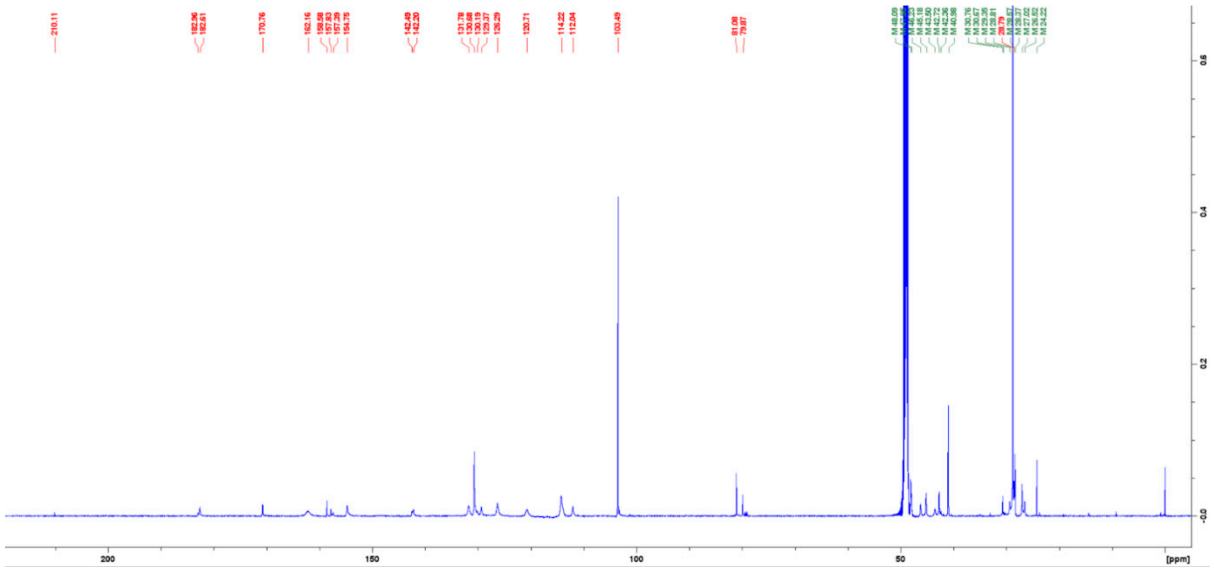

Boc-protected FITC probe **8b**

^{1}H-NMR

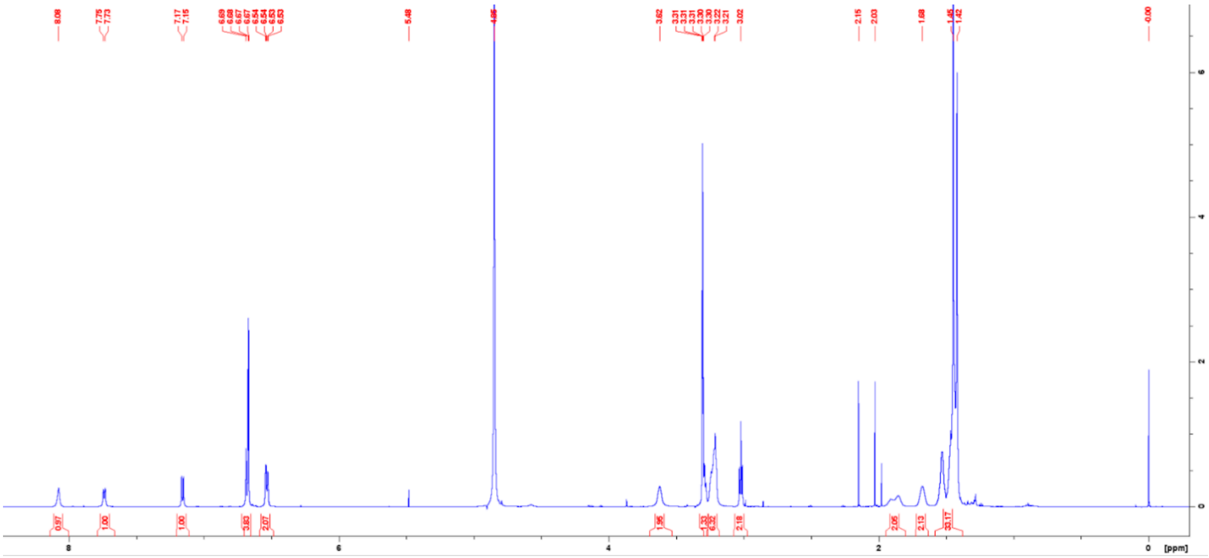

^{13}C-NMR

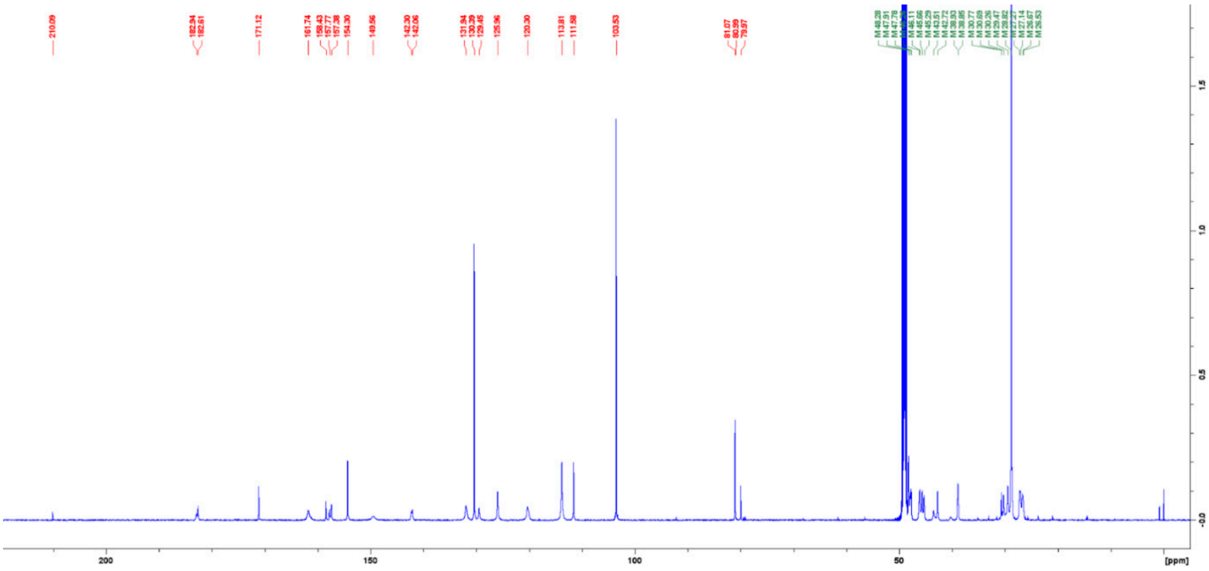

Boc-protected FITC probe **8c**

^{1}H-NMR

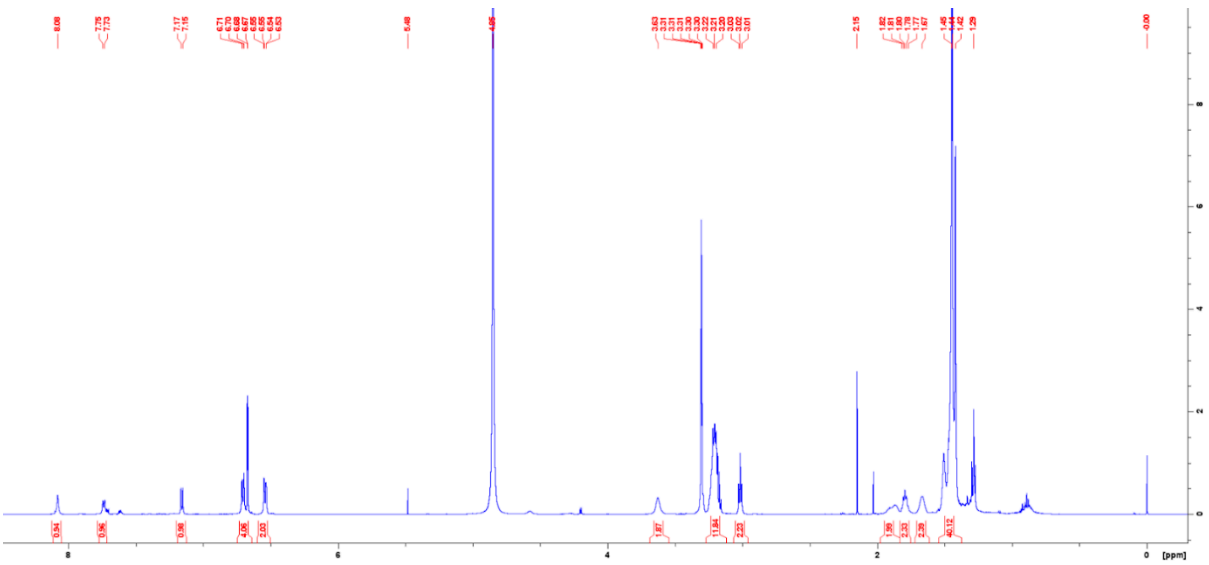

^{13}C-NMR

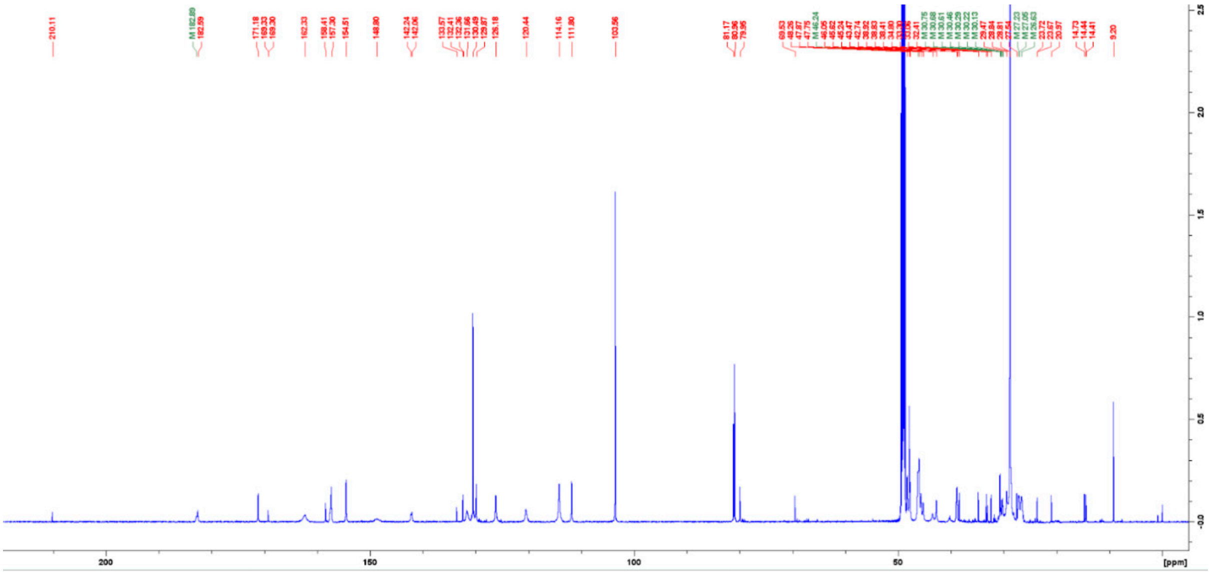

LC chromatograms and purity of final compounds

BODIPY-FL-T **2a**

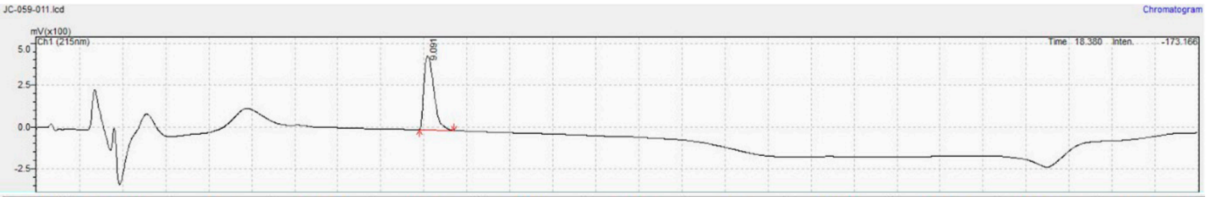

Peak#	Ret. Time	Area	Height	Mark	Conc.	Unit	ID#	Name	Area%	Conc.%	Norm. Conc.
1	9.091	7094078	441546	M	0.000				100.000	--	--
Total		7094078	441546		0.000				100.000		

BODIPY-FL-T **2b**

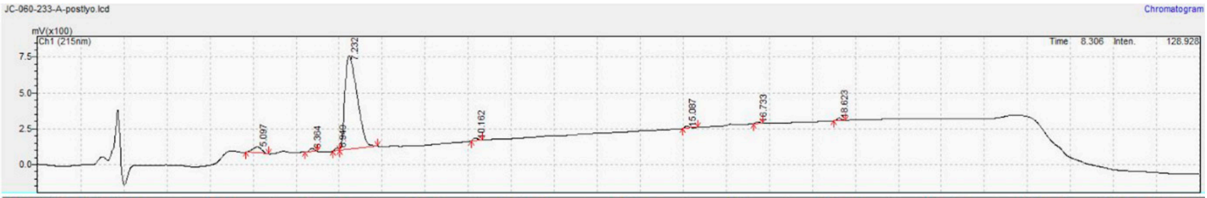

Peak#	Ret. Time	Area	Height	Mark	Conc.	Unit	ID#	Name	Area%	Conc.%	Norm. Conc.
1	5.097	647528	43248	M	0.000				4.527	--	--
2	6.364	193155	23412	M	0.000				1.350	--	--
3	6.949	125130	18666	M	0.000				0.875	--	--
4	7.232	12935679	654721	V M	0.000				90.428	--	--
5	10.162	111796	15064	M	0.000				0.782	--	--
6	15.087	93746	11890	M	0.000				0.655	--	--
7	16.733	79811	11430	M	0.000				0.558	--	--
8	18.623	118068	16693	M	0.000				0.825	--	--
Total		14304913	795125		0.000				100.000		

BODIPY-FL-T **2c**

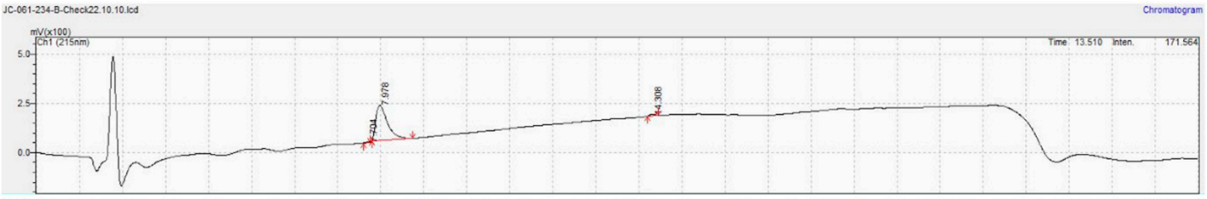

Peak#	Ret. Time	Area	Height	Mark	Conc.	Unit	ID#	Name	Area%	Conc.%	Norm. Conc.
1	7.704	15292	2141	M	0.000				0.455	--	--
2	7.978	3297839	177394	M	0.000				98.169	--	--
3	14.308	46223	5359	M	0.000				1.376	--	--
Total		3359354	184894		0.000				100.000		

BODIPY-FL-A **3a**

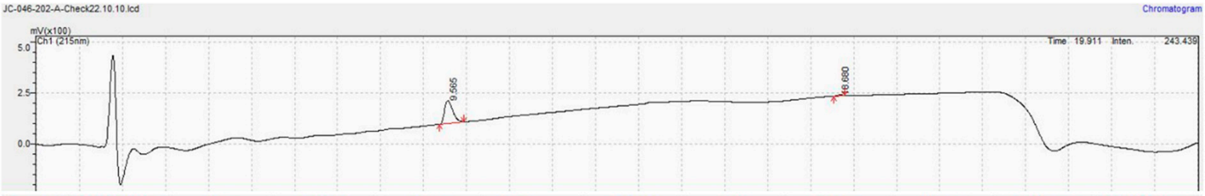

Peak#	Ret. Time	Area	Height	Mark	Conc.	Unit	ID#	Name	Area%	Conc.%	Norm. Conc.
1	9.565	1473154	111020	M	0.000				97.792	--	--
2	18.680	33262	4437	M	0.000				2.208	--	--
Total		1506417	115457		0.000				100.000		

BODIPY-FL-A **3b**

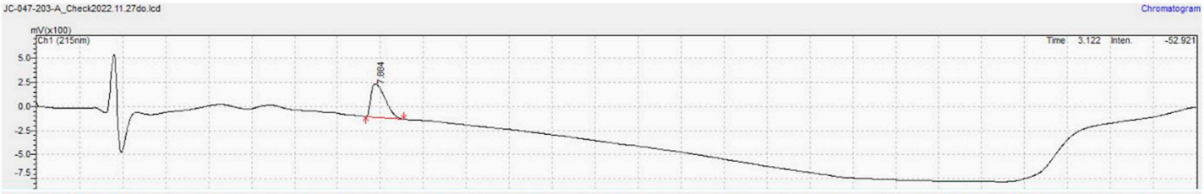

Peak#	Ret. Time	Area	Height	Mark	Conc.	Unit	ID#	Name	Area%	Conc.%	Norm. Conc.
1	7.884	7877180	347052	M	0.000				100.000	--	--
Total		7877180	347052		0.000				100.000		

BODIPY-FL-A **3c**

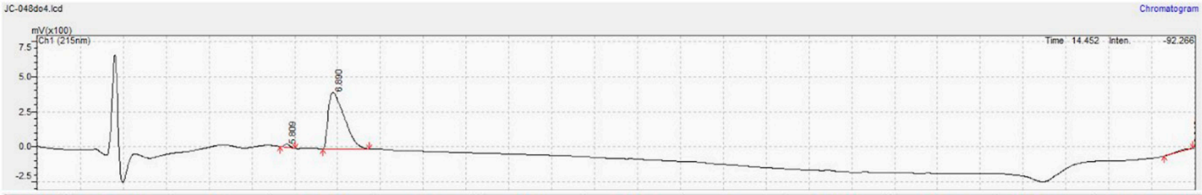

Peak#	Ret. Time	Area	Height	Mark	Conc.	Unit	ID#	Name	Area%	Conc.%	Norm. Conc.
1	5.809	224126	24367	M	0.000				2.031	--	--
2	6.890	10567139	404039	M	0.000				95.769	--	--
3	26.940	242678	2195		0.000				2.199	--	--
Total		11033943	430601		0.000				100.000		

FITC **4a**

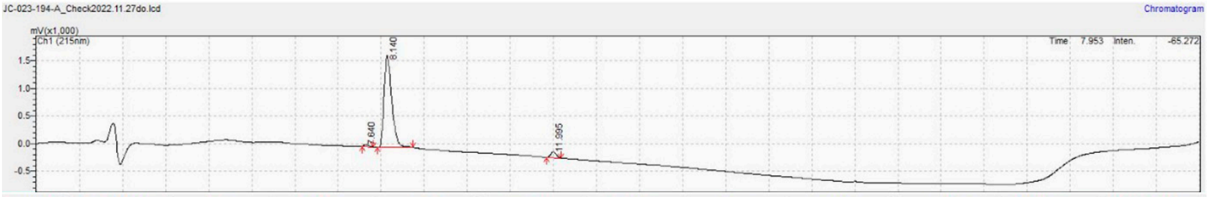

Peak#	Ret. Time	Area	Height	Mark	Conc.	Unit	ID#	Name	Area%	Conc.%	Norm. Conc.
1	7.640	266878	35899	M	0.000				1.219	--	--
2	8.140	20730211	1668711	M	0.000				94.699	--	--
3	11.995	893506	103654	M	0.000				4.082	--	--
Total		21890595	1808264		0.000				100.000		

FITC **4b**

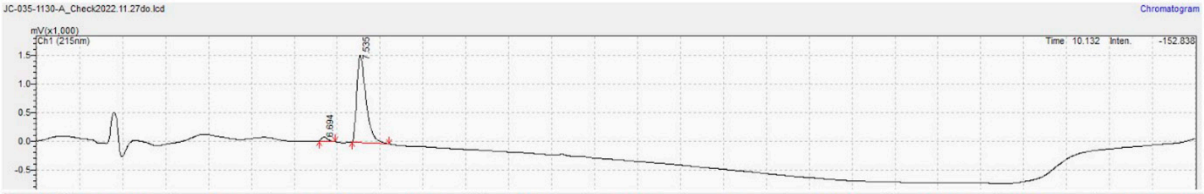

Peak#	Ret. Time	Area	Height	Mark	Conc.	Unit	ID#	Name	Area%	Conc.%	Norm. Conc.
1	6.694	743759	74920	M	0.000				3.128	--	--
2	7.535	23035879	1533920	M	0.000				96.872	--	--
Total		23779638	1608840		0.000				100.000		

FITC **4c**

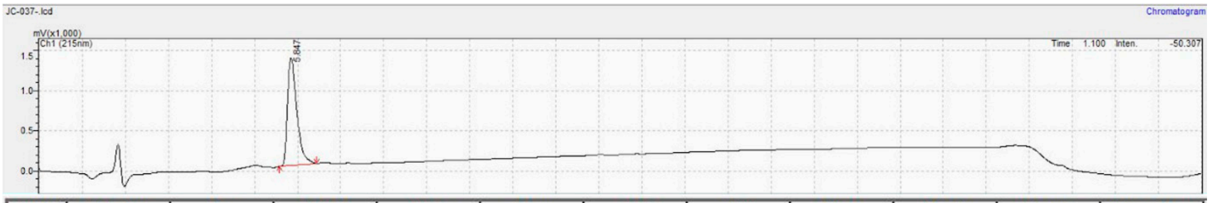

Peak#	Ret. Time	Area	Height	Mark	Conc.	Unit	ID#	Name	Area%	Conc.%	Norm. Conc.
1	5.847	20326612	1340541	M	0.000				100.000	--	--
Total		20326612	1340541		0.000				100.000		

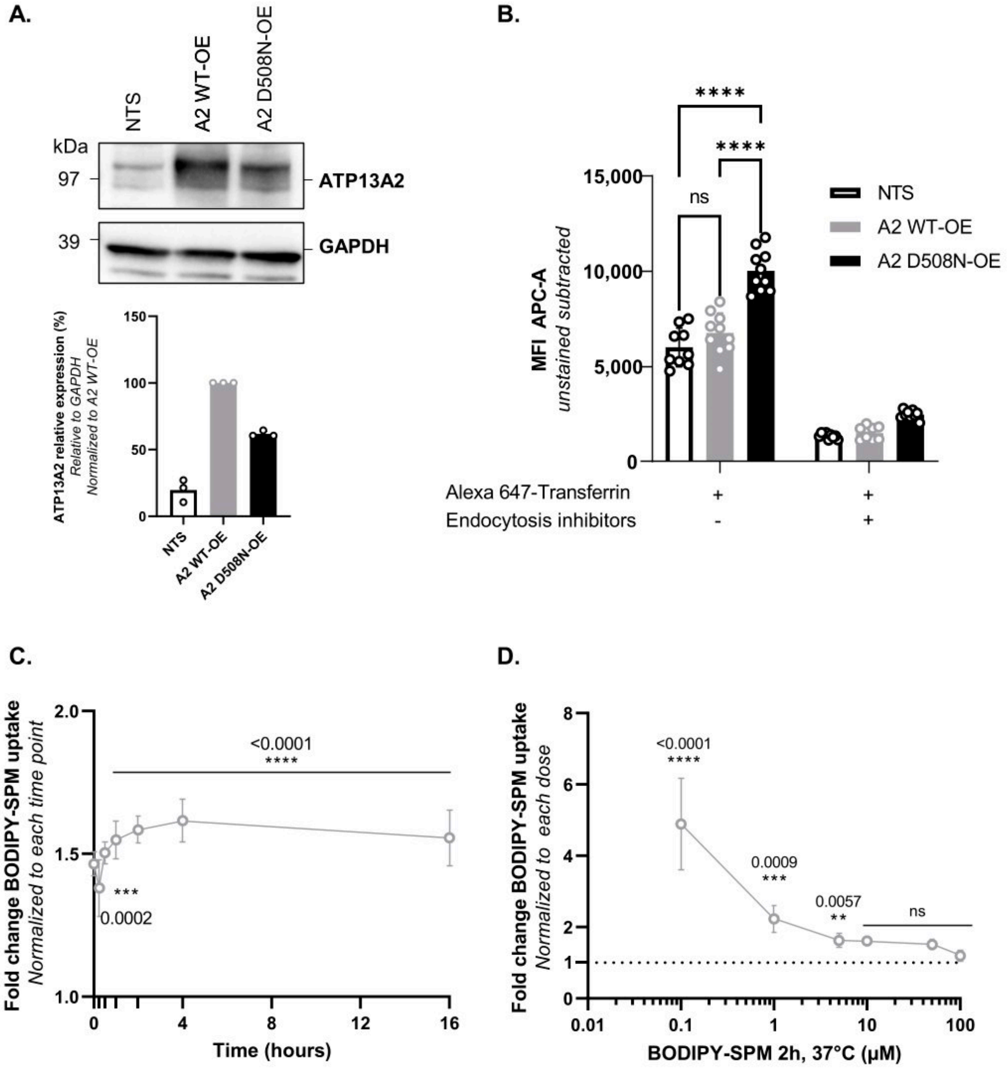

Figure S5. Characterization of ATP13A2-expressing SH-SY5Y cell models. (**A**). ATP13A2 protein expression level in SH-SY5Y cells transduced with lentiviral particles to overexpress ATP13A2 wild type (A2 WT-OE) or the catalytically dead mutant ATP13A2 D508N (A2 D508N-OE). Immunoblots showing ATP13A2 expression levels and quantification (bar graph) relative to GAPDH signal (N = 3). (**B**). The endocytosis rate in the ATP13A2 cell models was assessed by an Alexa 647-transferrin assay. A2 WT-OE, A2 D508N-OE and non-transduced (NTS) cells were incubated with Alexa 647-transferrin in the presence or absence of a cocktail of endocytosis inhibitors: dynasore (100 µM), genistein (50 µM) and pitstop2 (50 µM). The mean fluorescence intensity (MFI) of 10,000 events was recorded on the Canto II HTS flow cytometer (N = 3, two to four technical replicates, two-way ANOVA with Tukey's multiple comparisons test). In both panels (**A**,**B**), the NTS cell line was used as a control. (**C**,**D**). Graph depicts the fold change in BODIPY-SPM uptake between A2 WT-OE and A2 D508N-OE cells, normalized to each time point considered (**C**) or each dose considered (**D**) (N = 3 to 10, technical duplicates, two-way ANOVA with Sidak's multiple comparisons test; ns: not significant, **: $P < 0.01$; ***: $P < 0.001$; ****: $P < 0.0001$).

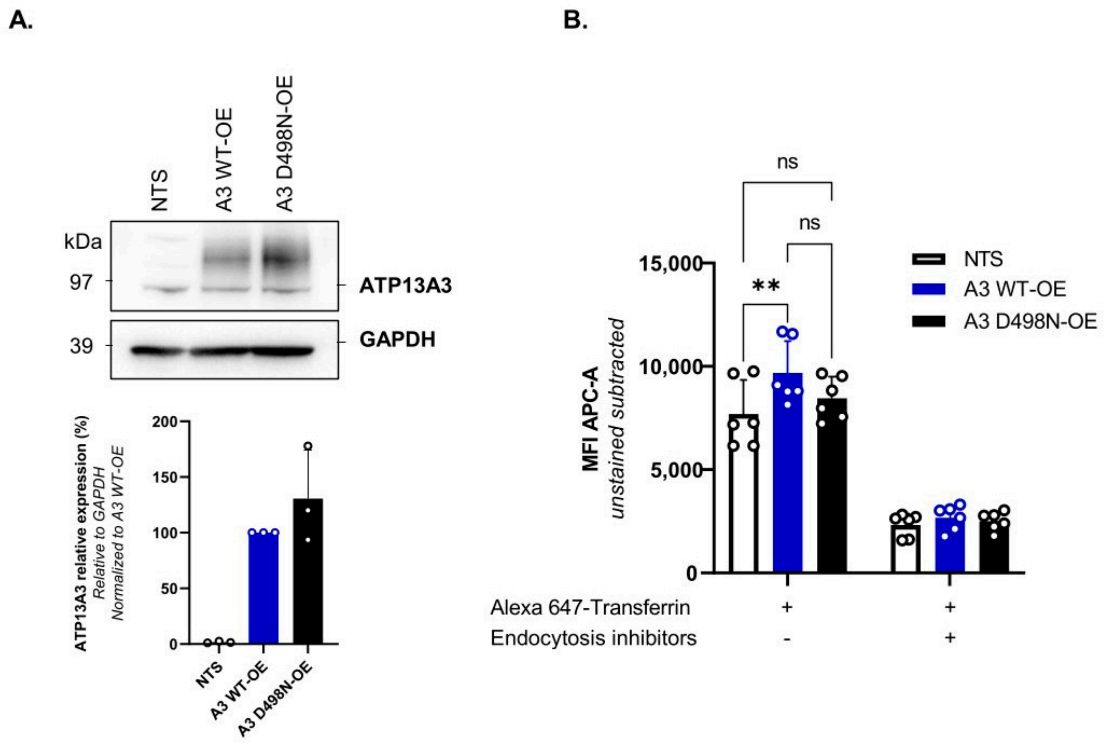

Figure S6. Characterization of ATP13A3-expressing HMEC-1 cell models. (**A**). HMEC-1 cells were transduced with lentiviral particles to overexpress ATP13A3 wild type (A3 WT-OE) or the catalytically dead mutant ATP13A3 D498N (A3 D498N-OE). Immunoblots showing ATP13A3 expression levels and quantification (bar graph) relative to GAPDH signal (N = 3). (**B**). Evaluation of the endocytosis rate in A3 WT-OE and A3 D498N-OE HMEC-1 cells. Cells were incubated with Alexa 647-transferrin in the presence or absence of a cocktail of endocytosis inhibitors: dynasore (100 μM), genistein (50 μM) and pitstop2 (50 μM). The mean fluorescence intensity (MFI) of 10,000 events was recorded on the Canto II HTS flow cytometer. N = 3 with technical duplicates, two-way ANOVA with Tukey's multiple comparisons test (ns: not significant, **: $P < 0.01$). In both panels, the non-transduced cell line (NTS) was used as a control.

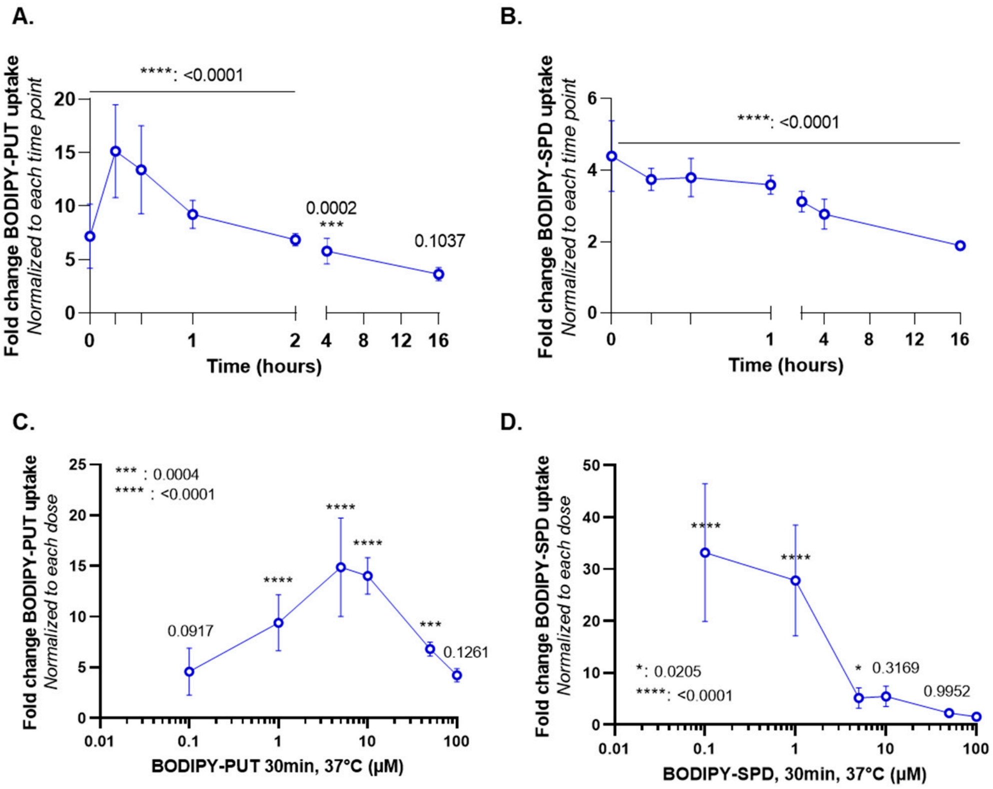

Figure S7. BODIPY-PUT and BODIPY-SPD uptake in HMEC-1 cells overexpressing ATP13A3 WT or D498N. Fold change in BODIPY-PUT (**A**,**C**) and BODIPY-SPD (**B**,**D**) uptake between A3 WT-OE and A3 D498N-OE cells, normalized to each time point considered (**A**,**B**) or each dose considered (**C**,**D**). N = 3 to 9, technical duplicates, two-way ANOVA with Sidak's multiple comparisons test ; ***: P < 0.001; ****: P < 0.0001.

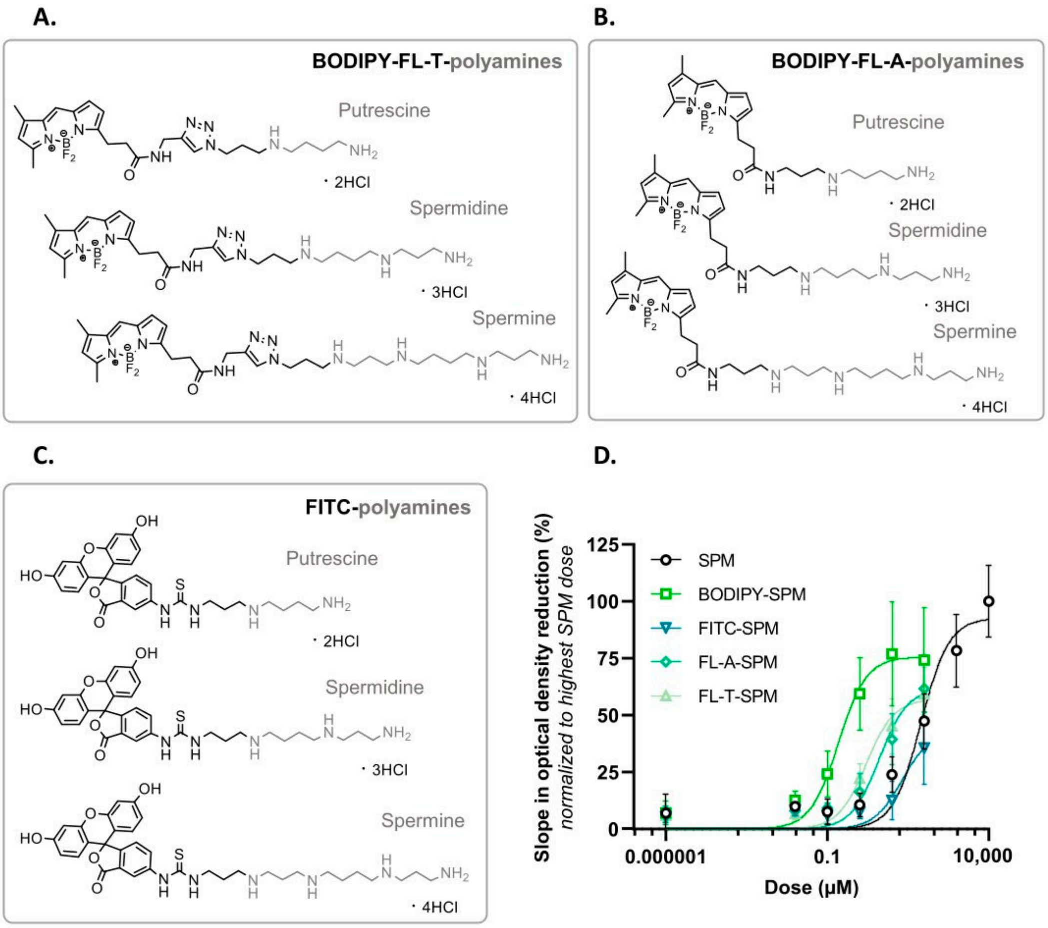

E.

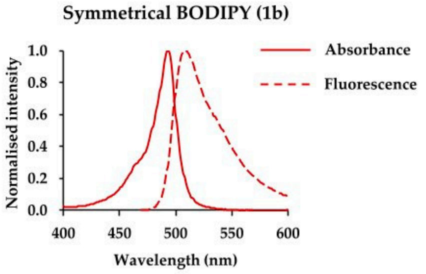

Compound	λ $_{abs}$ max (nm)	λ $_{Em}$ max (nm)	Stokes shift (nm)
**1b**	493	508	15
**2b**	502	511	9
**3b**	501	515	14
**4b**	488	518	30

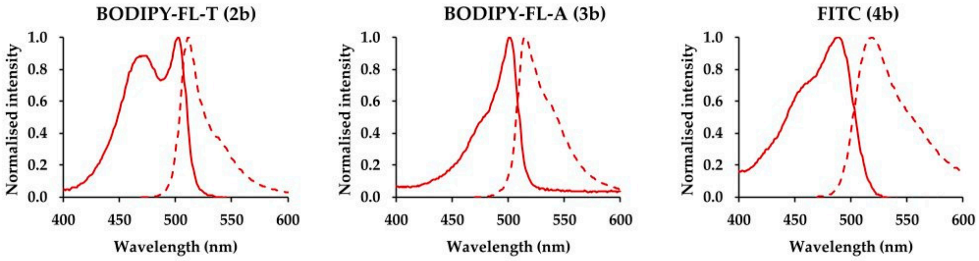

Figure S8. Full structures of the different green fluorescent polyamine conjugates used in this study and biochemical characterization of spermine conjugates. (**A**). FL-T-polyamine structures. (**B**). FL-T-polyamine structures. (**C**). FITC-polyamine structures. (**D**). Normalized absorption and emission spectra of symmetrical BODIPY-SPD (**1b**), BODIPY-FL-T-SPD (**2b**), BODIPY-FL-A-SPD (**3b**) and FITC-SPD (**4b**) together with a table providing the spectral properties of these compounds. (**E**). ATP/NADH-enzyme coupled ATPase assay. ATPase activity of purified ATP13A2 (1.25 μg) measured in the presence of increasing concentration of the following substrates: unlabeled spermine (SPM), BODIPY-SPM, FITC-SPM, FL-A-SPM and FL-T-SPM (N = 3 to 9, technical duplicates). The Y axis depicts the slope in optical density at 340 nm reflecting NADH consumption and further ATP consumption, normalized to the highest dose of SPM.

References Supplementary Information

1. Vanhoutte, R.; Kahler, J.P.; Martin, S.; van Veen, S.; Verhelst, S.H.L. Clickable Polyamine Derivatives as Chemical Probes for the Polyamine Transport System. *Chembiochem* **2018**, *19*, 907-911, doi:10.1002/cbic.201800043.
2. Hamouda, N.N.; Van den Haute, C.; Vanhoutte, R.; Sannerud, R.; Azfar, M.; Mayer, R.; Cortes Calabuig, A.; Swinnen, J.V.; Agostinis, P.; Baekelandt, V.; et al. ATP13A3 is a major component of the enigmatic mammalian polyamine transport system. *J Biol Chem* **2021**, *296*, 100182, doi:10.1074/jbc.RA120.013908.
